# Evidence for the Use of Triage, Respiratory Isolation, and Effective Treatment to Reduce the Transmission of *Mycobacterium Tuberculosis* in Healthcare Settings: A Systematic Review

**DOI:** 10.1093/cid/ciaa720

**Published:** 2020-06-05

**Authors:** Aaron S Karat, Meghann Gregg, Hannah E Barton, Maria Calderon, Jayne Ellis, Jane Falconer, Indira Govender, Rebecca C Harris, Mpho Tlali, David A J Moore, Katherine L Fielding

**Affiliations:** 1 TB Centre, London School of Hygiene & Tropical Medicine, London, United Kingdom; 2 Department of Health Services Research and Policy, London School of Hygiene & Tropical Medicine, London, United Kingdom; 3 University College Hospital, University College London Hospitals NHS Foundation Trust, London, United Kingdom; 4 Universidad Peruana Cayetano Heredia, Lima, Peru; 5 Hospital for Tropical Diseases, University College London Hospitals NHS Foundation Trust, London, United Kingdom; 6 Library and Archives Service, London School of Hygiene & Tropical Medicine, London, United Kingdom; 7 Centre for Infectious Disease Epidemiology and Research (CIDER), School of Public Health and Family Medicine, University of Cape Town, Cape Town, South Africa

**Keywords:** infection, prevention, LTBI, occupational health, healthcare worker

## Abstract

Evidence is limited for infection prevention and control (IPC) measures reducing *Mycobacterium tuberculosis* (MTB) transmission in health facilities. This systematic review, 1 of 7 commissioned by the World Health Organization to inform the 2019 update of global tuberculosis (TB) IPC guidelines, asked: do triage and/or isolation and/or effective treatment of TB disease reduce MTB transmission in healthcare settings?

Of 25 included articles, 19 reported latent TB infection (LTBI) incidence in healthcare workers (HCWs; absolute risk reductions 1%–21%); 5 reported TB disease incidence in HCWs (no/slight [high TB burden] or moderate [low burden] reduction) and 2 in human immunodeficiency virus-positive in-patients (6%–29% reduction). In total, 23/25 studies implemented multiple IPC measures; effects of individual measures could not be disaggregated.

Packages of IPC measures appeared to reduce MTB transmission, but evidence for effectiveness of triage, isolation, or effective treatment, alone or in combination, was indirect and low quality. Harmonizing study designs and reporting frameworks will permit formal data syntheses and facilitate policy making.

## Teaser

Twenty-five articles were found. Most studies showed reduced latent tuberculosis infection or tuberculosis disease incidence after implementation of an intervention package, but evidence was weak for effectiveness of individual interventions. We make recommendations for future research to provide a better evidence base.

**(See the Major Article by Fox et al on pages 15–26 and the Editorial Commentary by Griffith and Cegielski on pages 27–9.)**

Tuberculosis (TB) is the leading infectious cause of death worldwide [1, 2]. Healthcare workers (HCWs) are at higher risk of TB than the general population, likely because of exposure in health facilities [3–7]. Infection prevention and control (IPC) measures to reduce *Mycobacterium tuberculosis* (MTB) transmission in healthcare settings are considered under 3 categories: environmental controls (eg, mechanical ventilation), personal protection (eg, using respirators), and administrative controls (eg, coordinating efforts between governmental health departments) [8]. Evidence is limited, however, for the effectiveness of individual IPC measures in reducing MTB transmission, and guidelines have been written based heavily on expert opinion [9–11].

This systematic review was 1 of 7 complementary reviews commissioned by the World Health Organization (WHO) to inform the update of the 2009 TB IPC guidelines [12]. It aimed to answer the question: do (1) triage of people with TB signs, symptoms or with confirmed TB disease; and/or (2) respiratory isolation of presumed or demonstrated infectious TB cases; and/or (3) effective treatment of TB disease reduce the transmission of MTB to HCWs or other populations (including patients and visitors) in healthcare settings, when compared with transmission to the same populations in settings without, or with different, IPC interventions? The primary findings of this review were presented to the WHO guideline development group (GDG) and collated in an online appendix to the 2019 guidelines [13]. The guidelines contain recommendations for practice based on consideration of a wide range of evidence and should be the primary resource for implementation; this article looks more closely at how these interventions have been studied and discusses the implications for future TB IPC research.

## METHODS

The review protocol was registered on 12 February 2018 on the International Prospective Register of Systematic Reviews (ref. CRD42018085226) [14]. Countries were classified as high or low TB burden based on WHO lists published in 2016 [15].

### Population, Interventions, Comparators, and Outcomes

Populations of interest were HCWs and non-HCWs working in/attending healthcare settings with applied intervention/s. Interventions of interest, specified by the WHO GDG, were (1) triage based on signs, symptoms, or diagnosis of TB; (2) respiratory isolation (or spatial separation); and (3) effective treatment of TB based on bacteriologic susceptibility. WHO commissioned separate reviews to examine the use of environmental and personal protective measures [13]. Comparators used were HCWs and non-HCWs working in/attending healthcare settings with no or different intervention/s. Outcomes of interest were differences in latent tuberculosis infection (LTBI) or TB disease incidence/prevalence or measures of relative difference in incidence/prevalence (Appendix 1).

### Search Strategy, Terms, and Sources

Search strategies were constructed and run by an experienced professional librarian (final search 30 November 2017). Details of search terms and sources are provided in Supplementary Tables 1 and 2.

### Selection of Studies and Inclusion and Exclusion Criteria

Sifting (using criteria in Table 1) and data extraction were conducted in duplicate by 2 reviewers, with unresolved disagreements resolved by a third, independent reviewer, who also checked included articles. Citation tracking was conducted in Web of Science and/or Scopus® (details in Appendix 1). Systematic reviews meeting the inclusion criteria were used to find additional articles describing primary research and were not themselves included in the analysis.

**Table 1.** Inclusion and Exclusion Criteria Used During Sifting Process

Inclusion Criteria		Exclusion Criteria
Types of participants	Studies of:	1. Any study not in humans.
	1. HCWs (including CHWs) working in health care settings; or	2. Any study that did not report any of the above-stated outcomes of interest.
	2. Other staff working in a health care setting; or	3. Any study reporting solely on primary outcomes of interest without a control or comparator group.
	3. Persons of all ages (patients and visitors) attending health care settings, anywhere in the world.	4. Any systematic review superseded by an updated systematic review.
Types of intervention	At least 1 of the following:	5. Narrative reviews not adding new data or new analysis of data to existing knowledge.
	1. Triage of people with TB signs or TB symptoms or confirmed TB;	6. Commentaries and mathematical modelling studies.
	2. Respiratory isolation (spatial separation) of presumed infectious TB cases; or	7. Studies with fewer than 10 participants per comparator arm.
	3. Effective treatment of TB based on bacteriologic susceptibility.	8. Any study not written in English, Japanese, Chinese, Russian, French, Spanish or Portuguese.
Types of comparator	Studies reporting data (for outcomes of interest) from a control or comparator group of HCWs (including CHWs) working in health care settings, or other staff or persons of all ages (patients and visitors) attending health care settings, with no or different administrative infection control interventions.	9. Any study published before 1946.
Types of outcome measures	Studies reporting data on at least 1 of the outcome measures of interest (incidence/prevalence of LTBI or TB disease).	
Types of study	Any consecutive case series, case control study, cohort study, randomized controlled study, systematic review, or meta- analysis.	

Abbreviations: CHW, community health workers; HCWs, healthcare workers; LTBI, latent tuberculosis infection; TB, tuberculosis.

### Data Management and Assessment of Risk of Bias

Data management procedures are described in Appendix 1. Bias assessments were conducted at study level (using the Cochrane tool for experimental and prospective cohort studies [http://www.cochrane-handbook.org] and Downs & Black for other observational studies) [16] and at outcome level for Grading of Recommendations Assessment, Development, and Evaluation (GRADE [17, 18]; using scales for before/after studies [19] and cross-sectional studies [adapted Newcastle-Ottawa]).

### Data Analysis

Due to the heterogeneity of the data, study designs, and populations studied, meta-analysis could not be conducted. Findings were synthesized using a narrative approach, with studies organized in line with key outcomes of interest prespecified by WHO [14].

## RESULTS

**Figure 1.**
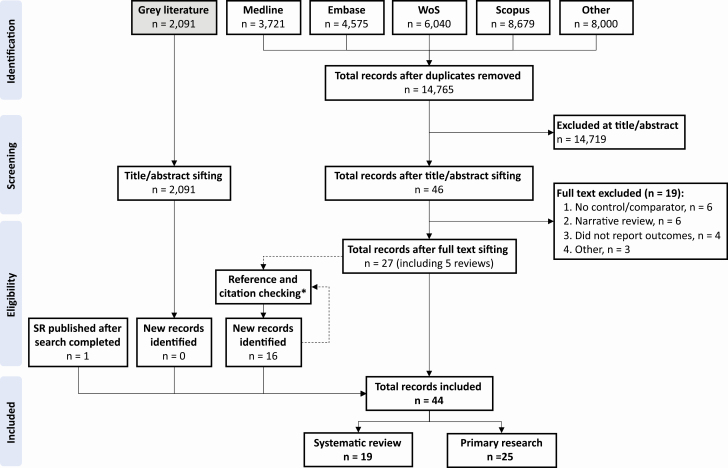
PRISMA flow diagram showing databases searched; numbers of records identified, sifted, reviewed, and included; and reasons for exclusion. *References and citations were checked for 25 primary research articles, 19 systematic reviews, and six guidelines (see Supplementary table 3). Abbreviations: PRISMA, preferred reporting items for systematic reviews and meta-analyses; Refs, references; SR, systematic review; WoS, Web of Science.

The search yielded 31 015 records; after removal of duplicates, 14 765 records were sifted by title and abstract (Figure 1). Forty-four articles were included: 25 primary research reports (Table 2) and 19 systematic reviews (Supplementary Table 3). Six TB IPC guidelines were also reviewed for possible primary research articles (Supplementary 3). Of the 25 studies, 17 (68%) were conducted in North America, 3 (12%) in sub-Saharan Africa, 2 (8%) each in Europe and Latin America, and 1 (4%) in East Asia; 19 (76%) were conducted in low TB burden (all high-income countries) and 6 (24%) in high TB burden settings (5 upper middle- and 1 low-income country); and 24 (96%) were conducted in hospitals and 1 (4%) in primary care facilities. Only 2 (8%) studies reported outcomes in non-HCWs attending healthcare facilities; in both cases these were human immunodeficiency virus (HIV)-positive in-patients. Nineteen (76%) studies described LTBI incidence, and 7 (28%) described TB disease incidence (1 described LTBI and TB disease incidence).

**Table 2.** Summary of Characteristics of Primary Research Studies Included (n = 25)

	Number of Studies Conducted		
Characteristic	Overall, n (%/25)	In Low Burden^a^ Countries, n (row %)	In High Burden^a^ Countries, n (row%)
All	25 (100)	19 (76.0)^b^	6 (24.0)^c^
Period conducted^d^			
Pre-1990	2 (8.0)	2 (100)	0
1990–1999	17 (68.0)	15(88.2)	2 (11.8)
2000–2009	5 (20.0)	2 (40.0)	3 (60.0)
2010 and later	1 (4.0)	0	1 (100)
Study design			
Cross-sectional	5 (20.0)	2 (40.0)	3 (60.0)
Before/after	12 (48.0)	10 (83.3)	2 (16.7)
During/after	8 (32.0)	7 (87.5)	1 (12.5)
Level of facility			
Primary	1 (4.0)	0	1 (100)
Secondary/tertiary	24 (96.0)	19 (79.2)	5 (20.8)
Group/s studied			
HCWs	23 (92.0)	17 (73.9)	6 (26.1)
Other individuals	2 (8.0)	2 (100)	0
Interventions implemented^e^			
Triage	15 (60.0)	11 (73.3)	4 (26.7)
Isolation	24 (96.0)	18 (75.0)	6 (25.0)
Effective treatment	5 (20.0)	5 (100)	0
Outcomes measured^**f**^			
LTBI incidence^g^	19 (76.0)	16 (84.2)	3 (15.8)
TB disease incidence^g^	7 (28.0)	3 (42.9)	4 (57.1)

Abbreviations: HCWs, healthcare workers; LTBI, latent tuberculosis infection; TB, tuberculosis.

^a^Based on WHO 2016 definitions [15].

^b^Of the 19 studies in low burden countries, 17 (89%) were conducted in North America and 2 (11%) in Europe; all 19 were conducted in high-income countries (per World Bank classifications at the time of study) [20].

^c^Of the 6 studies in high burden countries, 3 (50%) were conducted in sub-Saharan Africa, 2 (33%) in Latin America, and 1 (17%) in East Asia; 5 (83%) were conducted in upper-middle income countries and 1 (17%) in a low-income country (per World Bank classifications at the time of study) [20].

^d^Based on last year of data collection.

^e^Interventions of interest only; several studies implemented more than 1 intervention of interest.

^f^One study estimated incidence of both LTBI and TB disease.

^g^Generally reported as a risk or incidence rate.

**Figure 2.**
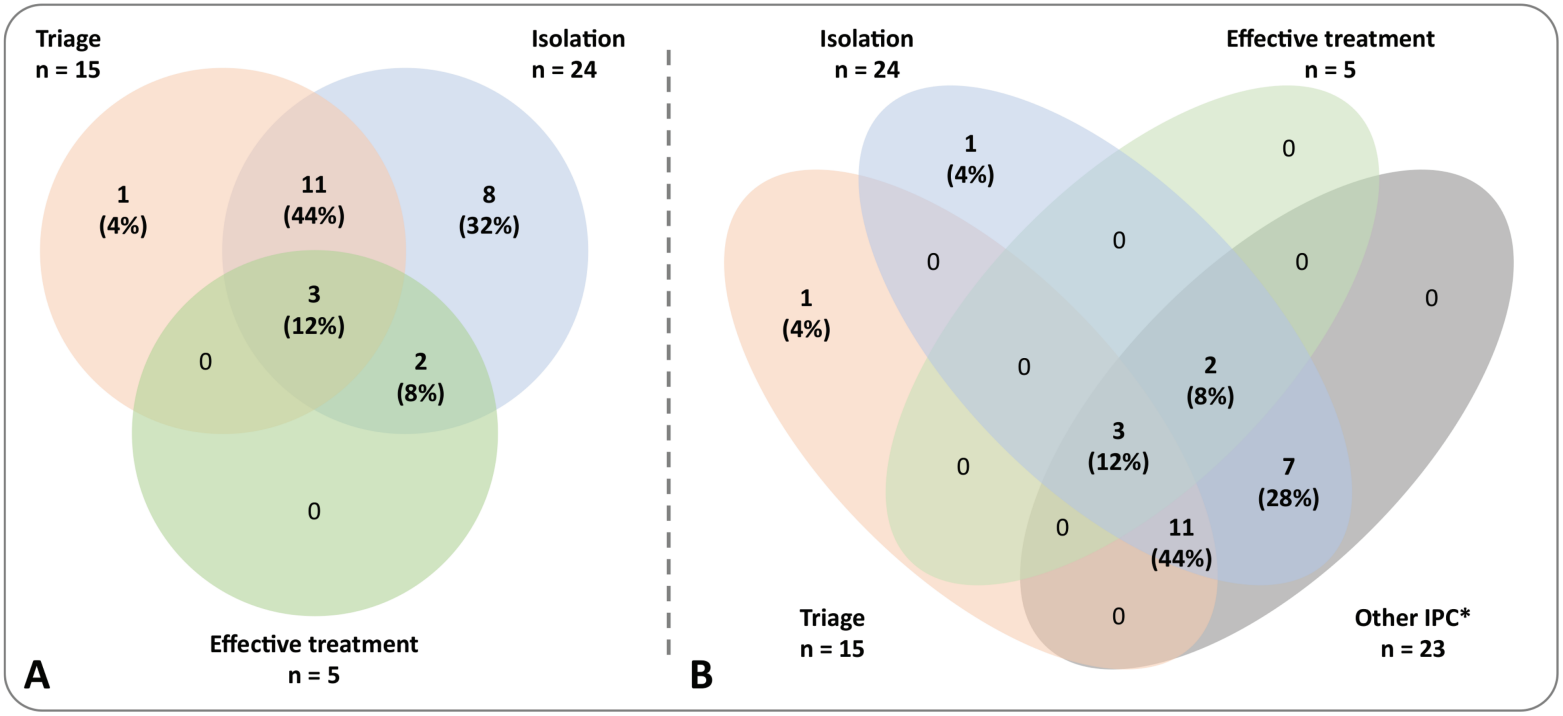
Venn diagrams showing overlap between interventions implemented in the 25 studies included. *A*, Overlap between the 3 interventions of interest. *B*, Overlap between the 3 interventions of interest and other IPC measures implemented. *Includes administrative, personal protective, and environmental IPC measures. Figure developed using Venny v2.1 [23], Inkscape (https://inkscape.org/), and GIMP (https://www.gimp.org/) software. Abbreviation: IPC, infection prevention and control.

Sixteen (64%) of 25 studies implemented interventions of interest in combination: 11 triage and isolation; 2 isolation and effective treatment; and 3 triage, isolation, and effective treatment (Figure 2A). Of the remainder, 8 (32%) studies assessed isolation alone, and 1 (4%) assessed triage alone. An obstacle to the evaluation of the 3 IPC interventions of interest was the paucity of studies that introduced only these interventions: all studies, except 2 [21, 22], implemented any or all of the 3 interventions as part of a wider suite of measures, including personal protective equipment (PPE) for HCWs; changes to ventilation and other environmental controls; and broader administrative controls (Table 3; Figure 2B). Disaggregation of the effects of individual measures was not possible, and it was therefore not feasible to attribute the entire reported effect on outcomes to a single intervention or to estimate the proportion of a demonstrated effect that could be attributed to the intervention (whether 1, 2, or all 3 elements of interest).

**Table 3.** Setting, Design, Population(s) Studied, Intervention(s) Implemented, and Outcome(s) Measured in the Studies Included, Divided by High/Low TB Burden^a^ Countries and Listed in Reverse Chronological Order of Publication (n = 25)

First Author, Year Published	Setting	Study Design	Population/s	Intervention/s		Comparator	Outcome (Metric Reported | How Measured)
				Administrative	Other		
High burden^a^							
O’Hara, 2017 [24]	28 public hospitals, South Africa	Cross- sectional	HCWS: all individuals working at study hospitals	TB infection control audit in 2012; separate score for administrative controls (triage, isolation).	Scores also for (i) environmental controls; (ii) personal respiratory protection; and (iii) miscellaneous measures, as well as overall score.	Facilities with higher vs those with lower administrative scores (a higher score equalled more IPC measures in place).	TB disease incidence (Episodes of TB disease per 100 000 HCWs | Ascertained through review and probabilistic matching of HR and TB register).
Claassens, 2013 [25]	121 primary health care facilities, South Africa	Cross- sectional	HCWs: all individuals employed at study facilities	TB infection control audit in May–Sep 2009; separate score for administrative controls.	Scores also for (i) environmental controls and (ii) personal respiratory protection, as well as overall score.	Facilities with higher vs those with lower administrative scores (a higher score equalled more IPC measures in place).	TB disease incidence, Jan 2006–Dec 2008 (Binary outcome defined as ≥1 TB episode among HCWs in a facility vs 0 episodes | Ascertained through questionnaire answered by facility manager).
da Costa, 2009 [26]	One hospital, Brazil	During/ after	HCWs: admin clerks, housekeepers, lab/radiology techs, nurses, physicians, social workers	Isolation of:all patients with sputum sent for AFB +/− mycobacterial culture, patients with productive cough until 1 smear negative, and HIV+ patients with abnormal CXR; and Education of HCWs.	Specialised TB o/p clinic. Use of N95 respirator for all person entering room with isolated patient. Patients leaving room for diagnostic tests wore surgical mask and educated on cough etiquette.	Period after (2002–2003) vs period during (1998–2001) implementation of IPC measures.^b^	LTBI incidence (TST conversions per 1000 PM | Annual serial TST, positive defined as ≥10 mm induration, conversion as ≥10 mm induration if initial 2-step or ≥15 mm if initial 1-step).
Roth, 2005 [27]	Four general hospitals, Brazil	Cross- sectional	HCWs: administrative workers, auxiliary staff, nurses, and physicians	Rapid diagnosis, treatment, and isolation.	Some hospitals had engineering measures (negative pressure isolation rooms with HEPA filtration and 20 ACH; N95 respirator for HCWs and surgical mask for patient until isolated.	Compared all 4 hospitals and 2 hospitals (A and B) with better IPC vs 2 hospitals (C and D) with worse IPC measures.	LTBI prevalence and LTBI incidence (Prevalence of positive TST and TST conversions per 1000 PM | Induration ≥10 mm, if <10 mm, TST repeated after 7–10 days).
Yanai, 2003 [28]	One referral hospital, Thailand	Before/ after	HCWs: no further details provided	Interventions (SOPs and IPC plan) aimed at: time from admission to initiation of TB treatment; timeliness of suspicion of TB; collection of specimens; reporting results, isolation, and initiation of treatment monitored.	Prevention interventions for HCWs and patients; engineering control measures (negative and natural ventilation; in lab, air exhaust and UVGI) and personal respirators (N95 masks encouraged when HCWs exposed to infectious TB patients. Lab staff processing MTB cultures used personal respirators).	Period after (1998–1999) vs period before (1995–1997) implementation of IPC measures.	LTBI incidence rate and TB disease incidence (TST conversions per 1,000 PY and TB disease incidence per 100 PY | Annual 2-step TST screening, positive defined as induration ≥10 mm; CXR, sputum smear and culture for all HCWs with symptoms or signs of active TB, those on treatment entered into HCW-specific register).
Harries, 2002 [29]	40 hospitals, Malawi	Before/ after	HCWs: all hospital- based staff with frequent exposure to medical patients	Guidelines on TB IPC (mid- 1998), including prioritising those with cough; rapid collection of sputum; frequency of processing specimens; and spatial separation of people with possible PTB.	Ventilation (windows left open) and masks (worn by TB patients when undergoing surgery).	Period after (1999) vs period before (1996) implementation of IPC measures.	TB disease incidence (Number/proportion of HCWs registered with TB | Consultation of administrative records and interviews with TB officers; details published separately [30]).
Low burden^a^							
Welbel, 2009 [31]	One public hospital, USA	During/ after	HCWs: nonclinical and clinical staff, including physicians, nurses, and training medical staff.	Creation of respiratory isolation service for proper and prompt isolation of patients; dedicated technician for service, collection of sputum daily. All HIV+ patients with respiratory symptoms placed in isolation. Implemented 1992–1997.	Engineering changes (negative pressure, UV lights); N95 respirators introduced in 1997.	Period after (1998–2002) vs period during (1994–1997) implementation of IPC measures.	LTBI incidence (TST conversions per 100 PY | Institutional 2-step TST programme established per CDC recommendations; positive defined as induration ≥10 mm).
Baussano, 2007 [32]	Three health units, Italy	Before/ after	HCWs: clerical, nursing, medical, and SW with negative TST and no previous vaccination with BCG.	Implementation of regional guidelines. Administrative: appointment of TB official at each facility; adoption of procedures to assess risk of TB transmission; prompt diagnosis and isolation of potentially infectious TB cases.	Organisational, technical and educational interventions; respiratory protection measures, particularly while performing cough-inducing procedures.	Period after (2002–2004) vs period before (1998–2000) implementation of IPC measures.	LTBI incidence (TST conversions per 100 PY, sex and age-adjusted | Positive defined as induration of ≥10 mm after previous negative TST).
Jones, 2002 [33]	One teaching medical centre, USA	During/ after	HCWs: employees of the medical centre (no other details provided).	Rule-out negative pathway: (1) initiation of respiratory isolation protocols; (2) direct patient admission/transfer to Special Immunology/Infectious Disease [SI/ID] unit; and (3) immediate patient placement in respiratory isolation.	Isolation rooms designed to provide negative pressure, six air exchanges/ hour, and venting of air outside.	Period after (Jan-Jun 1998) vs period during (1994–1998) the implementation of the pathway.	LTBI incidence (TST conversions | Review of employee health records).
Moro, 2000 [34]	One HIV ward in 1 hospital, Italy	Before/ after	HIV+ individuals admitted to outbreak ward whose stay overlapped with infectious periods of MDR-TB patients.	Strict AFB isolation procedures initiated for all patients with respiratory disease or fever.	Patients wore surgical masks when being transported for diagnostic purposes. Surgical masks mandatory for persons entering pts rooms.	Period after (Jul 1993–Feb 1994) vs before (Oct–Jun 1993) the implementation of IPC measures.	MDR-TB disease incidence (New cases per 1000 PD | case definition: signs, symptoms, and an isolate resistant to at least 1 first-line drug; medical and microbiology records consulted to ascertain history and drug susceptibility).
Bangsberg, 1999 [35]	One tertiary referral centre, USA	During/ after	HCWs: medical house staff.	All patients known HIV+, with HIV risk factors, or homelessness presenting with pneumonia/evidence of TB isolated on presentation at ED; admitted to negative-pressure isolation room; and remained in respiratory isolation until 3 negative AFBs.	Implementation of revised policy in 1992 based on CDC guidelines (published in 1993). Modifications to facility and personal protective equipment.	Period after (Dec 1992–Jun 1994) vs during (Jun 1992) the implementation of IPC measures.	LTBI incidence (TST conversions per 100 PY | Positive defined as induration of ≥10 mm, conversion as increase of ≥6 mm to a value of at least 10 mm).
Behrman, 1998 [36]	ED in 1 hospital, USA	Before/ after	HCWs: all employees at study hospital	New TB control measures in the ED, including 4 respiratory isolation rooms.	100% nonrecirculated air in trauma area, improved ventilation, laminar flow of air from registrars to patients, and acrylic plastic droplet shields for registrars.	Period after (1996) vs period before (Jul 1994–Dec 1995) implementation of IPC measures.	LTBI incidence (TST conversions | Positive defined as induration of ≥5 mm after 48–72 hours).
Blumberg, 1998 [37]	One public hospital, USA	During/ after	HCWs: staff in a teaching program (50% of their clinical rotations in the hospital)	Mandatory isolation of all patients with active TB, those with TB from differential diagnosis (or when sputum tests ordered), HIV+ or at high risk of HIV infection. Isolation discontinued after 3 consecutive negative AFBs/patient discharged.	Interim engineering controls (conversion of 90 rooms to negative pressure rooms by addition of window fan); personal respiratory protection equipment (submicron mask used by all HCWs entering respiratory isolation room.	Period after (Jan 1993–Jun 1997) vs period during (Jul–Dec 1992) implementation of IPC measures.	LTBI incidence (TST conversions per 100 PY | One-step testing; positive defined as induration of ≥10 mm after 48–72 hours).
Louther, 1997 [38]	Urban hospital with a dedicated “AIDS centre,” USA	During/ after	HCWs: All employees at study hospital (excluding those with boosted response to serial TST)	Respiratory isolation of all individuals suspected of having active TB; Triage of people attending ED/OPD and isolation of HIV+ people with particular symptoms (details published separately [39]).	Negative-pressure ventilation rooms.	Period after (1993–1994) vs period during (1991–1992) implementation of IPC measures.	LTBI incidence (Percentage TST conversions | Positive defined as induration ≥10 mm within 2 years of a previous negative result).
					Germicidal UV (n = 125 units) in patient rooms, waiting areas, and nursing stations.		
					PPE: Technol shield masks, dust- mist-fume respirators, and HEPA respirators.		
Uyamadu, 1997 [22]	One teaching hospital, USA	Before/ after	HCWs: all staff at study hospital	Mandatory respiratory isolation of all patients with community-acquired pneumonia, until 2 negative AFBs/TB ruled out on clinical grounds.	None	Period after (1988–Jul 1991) vs period before (Jul 1991–1994) implementation of isolation.	LTBI incidence (Percentage TST conversion | Positive defined as induration ≥10 mm after 48–72 hours).
Sinkowitz, 1996 [40]	1,494 hospitals, USA	Cross-sectional	Bronchoscopists and other HCWs	Compliance with 1990 CDC TB guideline AFB isolation room (survey conducted in March 1993, 50% response rate).	All 4 criteria; at least 3 criteria (negative-pressure, exhaust directed outside and single/cohorting of patients); at least negative-pressure criterion; at least the direct outside exhausted air criterion.	Hospitals implementing all 4 CDC criteria vs those not implementing all 4 criteria.	LTBI incidence (TST conversions measured in 1992, stratified by number of TB patients hospitalised in 1992 | Hospitals reported on proportion of HCWs with TST ≥10 mm and previous negative result).
Blumberg, 1995 [41]	One public hospital, USA	During/ after	HCWs at the hospital (not those on rotation)	Mandatory isolation of all patients with active TB, those with TB from differential diagnosis (or when sputum tests ordered), HIV+ or at high risk of HIV infection. Isolation discontinued after 3 consecutive negative AFBs/patient discharged.	Interim engineering controls and personal respiratory protection equipment (per Blumberg, 1998, above).	Period after (Jul 1992–Jun 1994) vs period during (Jan–Jun 1992) implementation of IPC measures.	LTBI incidence (TST conversions | One-step testing; positive defined as induration ≥10 mm after 48–72 hours).
Fridkin, 1995 [42]	210 hospitals, USA	Cross-sectional	HCWs, measured in 1992, among hospitals reporting at least 6 TB patients in 1992	Compliance with 1990 CDC TB guideline AFB isolation room (survey conducted in March 1993, 50% response rate); 1. All 4 criteria no vs yes (includes single/cohorting of patients); 2. ≥ 3 criteria (includes single/cohorting of patients).	Per Sinkowitz, 1996, above.	Hospitals implementing all 4 CDC criteria vs those not and hospitals implementing ≥3 CDC criteria vs those not.	LTBI incidence (TST conversions | reported percentage of HCWs who received a TST that became newly positive).
Holzman, 1995^c^ [43]	1 municipal hospital, USA	Before/ after	Nonphysician HCWs: nursing, housekeeping, radiology, and other staff	Implementation of 1990 CDC guidelines. Triage; early isolation and treatment (drug-susceptibility testing not specified); written criteria for starting/stopping precautions.	Negative pressure rooms with ventilation, filtration, and UV radiation equivalent to 28 air changes/hour; PPE: dust-mist and HEPA respirators.	Period after (Nov 1993–Oct 1994) vs period before (Nov 1992–Oct 1993) implementation of IPC measures.	LTBI incidence (TST conversions | Not described, cite CDC 1994 guidelines [44]).
Jarvis, 1995 [45]	Three hospitals (A[1989–91], B [1989–91] and D [1990–1]), USA	Before/ after	HCWs with baseline negative TST result and follow-up TST within 2 years	Implementation of 1990 CDC guidelines, including education of HCWs to increase index of suspicion for TB; prompt collection and processing of specimens; and prompt identification and isolation of pts with known/suspected TB.	Engineering controls (negative pressure isolation rooms and air exhausted outside) and respiratory protective devices (submicron or dust-mist).	Period after (not defined) vs period before (not defined) implementation of IPC measures.	LTBI incidence (TST conversions | Positive defined as induration ≥10 mm if unknown baseline or ≥10 mm increase on baseline induration).
Maloney, 1995 [46]	One teaching hospital, USA	Before/ after	HCWs with documented negative TST in previous 24 months	Implementation of 1990 CDC guidelines, including prompt isolation and treatment of patients with TB; rapid diagnosis.	Negative-pressure isolation rooms; moulded surgical masks for HCWs.	Period after (Jul 1991–Aug 1992) vs period before (Jan 1990–Jun 1991) implementation of IPC measures.	LTBI incidence (TST conversions| Positive defined as induration ≥10 mm).
Stroud, 1995 [47]	One hospital, USA	Before/ after	“AIDS patients” with same ward exposure to MDR-TB pts	Aggressive implementation of administrative controls: rapid placement of TB patients or suspected TB patients in single-patient rooms. Expanded TB treatment prescribed.	In period III, engineering changes (some isolation rooms fitted with UV lights and fans that exhausted air outside) provided ≥6 air exchanges/hour and created negative pressure in hallway.	Period after (Apr 1990–May 1991) vs period before (Jan 1989–Mar 1990) implementation of IPC measures.	MDR-TB disease risk (“Attack rate,” expressed as % | Case definition: diagnosis of active TB during study period with MTB isolate resistant to at least isoniazid and streptomycin, ascertained through review of hospital and medical records).
Wenger, 1995 [48]	One HIV ward in hospital, USA	Before/ after	HCWs working on the HIV ward	Higher index of suspicion for TB, stricter criteria for discontinuing isolation; restriction of cough procedures to isolation rooms; expansion of initial TB treatment to 4 agents; shorter turnaround time for AFB, DST.	Negative pressure, masks.	Period after (Jun 1990–Jun 1992) vs period before (Jan–May 1990) implementation of IPC measures.	LTBI incidence (TST conversions | Positive defined as induration ≥10 mm and ≥6mm larger than previously reported induration).
Bryan, 1983 [49]	One teaching hospital, USA	During/ after	HCWs (no details provided)	TB Registry: documents dates/results of AFBs, CXR results, and whether patient is put in respiratory isolation and when; register reviewed weekly by TB epidemiologist.	None	Period after (1977–1981) vs period during (1976) implementation of IPC measures.	LTBI incidence (TST conversion rates | 1-step testing; no other details provided).
Jacobson, 1957 [21]	One general hospital, USA	Before/ after	HCWs: hospital employees (before 1952, only physicians, student nurses and TB staff examined regularly)	Routine CXR screening programme for new admissions.	None	Period after (1952–1955) vs period before (1942–1951) implementation of screening programme.	TB disease incidence rate (TB cases per 1000 PY | Identified through CXR and/or clinical examination and/or sputum microscopy).

↑: increase; ↓: decrease.

Abbreviations: ACH, air changes per hour; adj., adjusted; admin, administrative; AFB, acid-fast bacilli; BCG, bacille Calmette-Guérin; CDC, Centers for Disease Control and Prevention; CI, confidence interval; CXR, chest x-ray; DST, drug sensitivity testing; ED, emergency department; HCWs, healthcare workers; HEPA, high efficiency particulate air; HIV, human immunodeficiency virus; HIV+: HIV-positive; HR, human resources; IPC, infection prevention and control; lab, laboratory; MDR, multidrug-resistant; MTB, Mycobacterium tuberculosis; mth, month; o/p, outpatient; OR, odds ratio; PD, person-day; PM, person-month; pt, patient; PY, person-years; SOP, standard operating procedure; SW, social worker; TB, tuberculosis; tech, technician; TST, tuberculin skin test; unadj., unadjusted; UV, ultraviolet; UVGI, ultraviolet germicidal irradiation.

^a^Based on WHO 2016 definitions [15].

^b^This paper refers to both 1998–2001 and 1999–2001 as the first period of observation; 1998–2001 used for the purposes of this review.

^c^Conference abstract only.

### Studies Implementing Triage of People With TB Signs and/or Symptoms

Fifteen studies implemented triage: 11 (73%) in low burden settings, all in secondary or tertiary health facilities. Definitions of triage varied widely, from screening of patients “with pneumonia or evidence of TB” [35] to an “expanded respiratory isolation policy” [37, 41]. The only study to use triage alone used “routine chest x-ray screening for all new admissions” [21].

Among 10 studies reporting changes in LTBI incidence (all implemented composite interventions), estimates of effect ranged from an absolute reduction of 2.3% (n = 21 197) [41] to 20.5% (n = 65; Table 4) [48]. Of the 4 studies reporting incidence rates (IRs) [28, 32, 37, 38], IR ratios ranged from 0.18 to 0.9 (unadjusted; some calculated).

**Table 4.** Main Findings of Included Studies Divided by High/Low TB Burden^a^ Countries and Listed in Reverse Chronological Order of Publication (n = 25)

First Author, Year Published	Country	Intervention/s				Outcome/s | Population/s	Primary Findings			Other Findings
		Tri^g^	Isol^n^	Tx	Oth^b^		No Intervention	Intervention	Effect Estimate	
High burden										
O’Hara, 2017 [24]	South Africa	Yes	Yes	No	Yes	TB disease | HCWs	Not reported	Not reported	Unadj. OR for higher vs lower administrative score 0.94 (95% CI .87–1.02), *P* = .12	• Median administrative score 21 (IQR 18–24, range 15–28); max possible score 32
									Adj. OR for higher vs lower administrative score 0.97 (95% CI .90–1.04), *P* = .36	• Adj. OR adjusted for environmental score, PPE score, miscellaneous score, and number of TB patients
Claassens, 2013 [25]	South Africa	No	Yes	No	Yes	TB disease | HCWs	Not reported	Not reported	Unadj. OR for higher vs lower administrative score (continuous): 1.09 (95% CI .99–1.19), *P* = .07	• Administrative score: range from −4 to 19, mean 8 (SD 4). Administrative score not included in adjusted model
										• ORs also shown for total, environmental controls, and personal respiratory protection scores
da Costa, 2009 [26]	Brazil	No	Yes	No	Yes	TST conversion rate | HCWs	1998–2001: 5.8 (95% CI 4.9–6.7) per 1,000 PM (25 events in 4,307 PM)	2002–2003: 3.7 (95% CI 2.8–4.6) per 1,000 PM (15 events in 3,858 PM)	Hazard ratio = 0.46 (95% CI .23–.89), *P* = .006	• Adjusted hazard ratio 0.24 (95% CI .10–.54), adjusted for exposure to person with PTB in hospital and occupation
										• Fidelity: reduced time between microscopy request and result between 2 time-periods
										• Increased proportion of PTB diagnosed among suspected cases isolated
Roth, 2005 [27]	Brazil	Yes	Yes	No	Yes	TST prevalence | HCWs	TST prevalence: Hosp C: 65.8% (574/872); Hosp D: 62.2% (454/730)	TST prevalence: Hosp A: 46.7% (407/872); Hosp B: 69.6% (1,353/1,945)	Hospital C and D, 16.0/1,000 PM vs A and B, 7.8/1,000 PM; *P* < .001. Hospitals B, C, and D vs A: unadj. OR 1.3, 3.2, and 3.4, respectively; adj. OR (95% CI; p-value) 1.0 (0.5–1.8; NS), 2.3 (1.2–4.2; 0.01), 2.8 (1.4–5.6; 0.002), respectively.	• Reported annual number of new PTB cases: Hosp A 200–250; Hosp B 100–150; Hosp C 450–500; and Hosp D 50–60
						TST conversion rate | HCWs	TST conversion: Hosp C: 19.8/1,000 PM (n = 34); Hosp D: 12.2/1,000 PM (n = 21)	TST conversion:Hosp A: 7.4/1,000 PM (n = 19); Hosp B: 8.1/1,000 PM (n = 31)		
Yanai, 2003 [28]	Thailand	Yes	Yes	No	Yes	TST conversion rate | HCWs	TST conversion 1995–1997: 9.3 (95% CI 3.3–15.3) per 100 PY	TST conversion 1998: 6.4 (95% CI 1.5–11.4) per 100 PY;	TST conversion Rate ratio (vs 1995–1997) 1998: unadj. 0.9 (0.4–2.2); adj. 0.4 (0.1–1.6), *P* = .2.	• Intervention implemented in 1996. Increase in numbers of smear-positive TB patients identified 1990 (102) to 1999 (356)
								1999: 2.2 (95% CI 0–5.1) per 100 PY	1999: unadj. 0.03 (0.01–0.2); adj. 0.01 (0–0.04), *P* < .001	• Numerators and denominators unclear
										• Active TB disease incidence among HCWs also reported for the period 1988–1994
						TB disease rate | HCWs	TB disease 1995–1997: 0.65 (29/4,464) per 100 PY	TB disease 1998: 0.42 (7/1,654) per 100 PY; **1999:** 0.44 (7/1,583) per 100 PY	TB disease No rate ratio reported	
Harries, 2002 [29]	Malawi	Yes	Yes	No	Yes	TB disease | HCWs	1996: 3.7% (100/2,697)	1999: 3.2% (96/2,979)	Reported as “nonsignificant”	• Fidelity (Jan–Jun 1998 vs Jan–Jun 1999): similar length of time from admission to diagnosis and treatment
Low burden										
Welbel, 2009 [31]	USA	Yes	Yes	Yes	Yes	TST conversion rate | HCWs	1994: 4.22/100 PY	1998: 0.74/100 PY;	2002 vs 1994: *P* < .001	• Number of inpatients with active TB declining from 1997 onwards
							1995: 2.92/100 PY;	1999: 0.57/100 PY;	1997 vs 1994: *P* < .001	
							1996: 1.41/100 PY;	2000: 1.04/100 PY;	2002 vs 1997: *P* = .14	
							1997: 1.48/100 PY	2001: 0.71/100 PY;		
								2002: 0.28/100 PY		
Baussano, 2007 [32]	Italy	Yes	Yes	No	Yes	TST conversion rate | HCWs	Jan 1998–Jun 2000: 2.19 (95% CI 1.81–2.56) per 100 PY (106 events in 4,034 PY)	Jan 2002–Dec 2004: 0.84 (95% CI .55–1.28) per 100 PY (42 events in 4,463 PY)	Not reported	• Events per PY includes data to Dec 2001.
										• Data also shown by occupation (work activity) and workplace
Jones, 2002 [33]	USA	No	Yes	No	Yes	TST conversion | HCWs		Overall (1995–1998): 2.3% (92/~4000); In SI/ID unit Jan 1994–Jan 1998: 0% (0/60); Feb–Jun 1998: 3.33% (2/60)	Not reported	• Fidelity: 50 patients placed on pathway from 1995–1998
Moro, 2000 [34]	Italy	Yes	Yes	No	Yes	MDR-TB incidence rate | HIV-positive in-patients	Oct–Jun 1993: 10.6/1,000 PD (26 events in 2,455 PD; 90 individuals exposed)	Jul 1993–Feb 1994: 0/1,000 PD (0 events in 654 PD; 44 individuals exposed)	Not reported	• 37 patients exposed in both before and after period: 0/1,839 MDR-TB episodes
										• Over the entire time period there were several infectious MDR-TB cases in the ward
Bangsberg, 1999 [35]	USA	No	Yes	No	Yes	TST conversion rate | HCWs	Jun 1992: 5.8/100 PY (9/88 [10.3%])	Dec 1992: 5.1/100 PY (2/77 [2.6%]); Jun 1993: 0/100 PY (0/88 [0%]); Dec 1993: 2.3/100 PY (1/93 [1.1%]); Jun 1994: 0/100 PY (0/86 [0%])	Not reported overall	• Fidelity: proportion properly isolated increased from 38% (Jan–Jun 1992) to 75% (Jul–Dec 1993)
									Data also reported for interns showing a decline over 1992–1994 (*P* = .029)	• Participation of house staff ranged from 77% to 88% in the years reported
Behrman, 1998 [36]	USA	Yes	Yes	No	Yes	TST conversion | HCWs	Jul 1994–Dec 1995 (cycle 2) ED staff: 12.0% (6/50) Other hospital employees: 2.1% (51/2,514)	1996 (cycle 3) ED staff: 0% (0/64) Other hospital employees: 1.2% (36/3,000)	Not reported	• No change in the number of culture-positive admissions from 1993–1996. No data reported on frequency of use of respiratory isolation rooms
										• No data on TST conversions from Mar 1993–Dec 1994 (cycle 1)
Blumberg, 1998 [37]	USA	Yes	Yes	No	Yes	TST conversion rate | HCWs	Jul–Dec 1992: 5.98/100 PY (21 conversions)	Jan 1993–Jun 1997: 1.09/100 PY (31 conversions)	Not reported, but reported a p-value comparing the 2 time-periods: <0.001	• Also showed data separately for US medical school graduate staff during and after implementation (5.26 vs 0.72/100 PY, *P* < .001; 17 and 19 conversions, respectively)
Louther, 1997 [38]	USA	Yes	Yes	No	Yes	TST conversion | HCWs	1991–1992 Overall 7.2 conversions per 100 PY (65 events in 898 PY) Lab workers 6.3 (3/48); Physicians/nurses 7.2 (26/363); Social service 8.1 (9/111); Housekeeping 11.7 (21/179); Finance 3.0 (6/197)	1993–1994 Overall 3.3 conversions per 100 PY (32 events in 971 PY) Lab workers 2.3 (1/44); Physicians/nurses 3.0 (12/398); Social service 2.2 (3/139); Housekeeping 6.7 (12/179); Finance 1.9 (4/211)	Overall crude rate ratio 0.46^c^; *P* = .001 Lab workers 0.37, *P* = .42; Physicians/nurses 0.42, *P* = .01; Social service 0.27, *P* = .04; Housekeeping 0.57, *P* = .12; Finance 0.63, *P* = .48	• Number of new cases of TB per year ranged from 56 to 118 over study period
										• Isolation days per year ranged from 6360 to 10 883 over study period
										• Information on TST available for >90% of all employees
Uyamadu, 1997 [22]	USA	No	Yes	No	No	TST conversion | HCWs	1988–1990: overall 0.6% (23/3,842);	Jul 1991–Dec 1994: average	Not reported	• Fidelity: 100% compliance with respiratory isolation
							Jan–Jul 1991: 1.7% (13/768).	Jul–Dec 1991: 1.3% (10/774)		
										• Number of new TB cases in hospital remained stable from 1991–1994
								1992: 0.5% (9/1,637)		
								1993: 0.7% (9/1,325)		
								1994: 0.6% (8/1,381)		
Sinkowitz, 1996 [40]	USA	No	Yes	No	Yes	TST conversion | Bronchoscopists	All 4 criteria—no: Bronchoscopists No TB pts: 0% (n = 16);	All 4 criteria—yes: Bronchoscopists No TB pts: 3.3% (n = 13);	Not reported	• Results also reported for negative-pressure, air exhaust, and respiratory protection criterion
							1–5 TB pts: 8.0%; (n = 22);	1–5 TB pts: 8.3% (n = 39);		
							≥6 TB pts: 5.1% (n = 11)	≥6 TB pts: 5.7% (n = 16)		
						TST conversion | HCWs	HCWs No TB pts: 0.49% (n = 127);	HCWs No TB pts: 0.53% (n = 75);		
							1–5 TB pts: 0.64% (n = 116);	1–5 TB pts: 0.69% (n = 185);		
							≥6 TB pts 0.76% (n = 34)	≥6 TB pts 0.90% (n = 66)		
Blumberg, 1995 [41]	USA	Yes	Yes	No	Yes	TST conversion | HCWs	Jan–Jun 1992: 3.3% (118/3579)	Jul–Dec 1992: 1.7% (51/2,975);	Not reported	• Fidelity to the intervention: Jul 1991–Feb 1992 (8 mths) 4.4 TB exposure episodes/mth (35/103 not appropriately isolated) vs Mar 1992–Jun 1994 (28 mths) 0.6 TB exposure episodes/mth (18/358 not appropriately isolated)
								Jan–Jun 1993: 1.4% (67/4,715);	Reported P-value comparing the 5 time-periods: <.001	
								Jul–Dec 1993: 0.6% (30/4775);		
								Jan–Jun 1994: 0.4% (23/5,153)		
Fridkin, 1995 [42]	USA	No	Yes	No	Yes	TST conversion | HCWs	All 4 criteria—No: 1.89% (383/20 296; 17 hospitals).	All 4 criteria—Yes: 0.60% (348/57 600; 28 hospitals).	All 4 criteria: Yes vs No: *P* = .02; ≥3 criteria: Yes vs No: *P* = .03	• Similar trend for at least negative-pressure or at least the direct outside exhausted air criterion
										• Also restricted analysis to high risk HCWs (includes bronchoscopists and respiratory therapists) and found similar results
							≥3 criteria—No:1.83% (380/20 776; 16 hospitals).	≥3 criteria—Yes:0.62% (376/60 371; 30 hospitals).		
Holzman, 1995 [43]^d^	USA	Yes	Yes	No	Yes	TST conversion | HCWs	Nov 1992–Oct 1993: Overall 90/2,132 (4.2%);	Nov 1993–Oct 1994 Overall 23/1,995 (1.2%);	Percentage reduction (95% CI), *P*-value	
							Nursing 54/608 (8.9); Housekeeping 9/105 (8.6);	Nursing 11/519 (2.1); Housekeeping 3/90 (3.3); Radiology	Overall 73% (57–43), *P* < .001;	
							Radiology 2/50 (4.0);	1/74 (1.4); Misc./Unk.	Nursing 76% (44–90), *P* < .001;	
							Misc./Unk. 14/474 (3.0)	1/573 (0.2)	Housekeeping 61% (0–89); Radiology 66% (0–97); Misc./Unk. 94% (55–99), *P* < .001	
Jarvis, 1995 [45]	USA	No	Yes	Yes	Yes	TST conversion | HCWs	Baseline period (not defined):	Intervention period (not defined)	A: *P* = .01;	• Fidelity: proportion of patients on ward with same-ward exposures decreased in intervention (15%) vs baseline period (74%). Decreased in all hospitals
							A: 24% (7/29);	A: 0% (0/23);		
							B: 9% (2/22);	B: 18% (6/33);	B: *P* = NS;	
							D: 12% (15/123)	D: 3% (5/150)	D: *P* = .01	
										• In hospital B there was incomplete implementation of CDC guidelines
Maloney, 1995 [46]	USA	No	Yes	Yes	Yes	TST conversion | HCWs	Jan 1990–Jun 1991	Jul 1991–Aug 1992		• TST conversion data also reported subgroup direct/no direct patient contact
							Overall: 3.1% (26/840)	Overall: 3.0% (22/727)	Overall: *P* = .9	
							Wards housing TB patients: 16.7% (15/90);	Wards housing TB patients: 5.1% (4/78);	Wards housing TB patients: relative risk = 3.2, *P* = .02;	• Fidelity: AFB isolation before 40% vs after 90%; receiving adequate treatment before 43% vs after 90%
							Other wards: 2.8% (7/254)	Other wards: 4.0% (9/228)	Other wards: relative risk = 0.7, *P* = .5	
Stroud, 1995 [47]	USA	Yes	Yes	Yes	Yes	MDR-TB risk | AIDS patients	Jan 1989–Mar 1990 (period 1): 8.8% (19/216)	Apr 1990–May 1991 (period 2): 2.6% (5/193)	*P* = .01	• Period 1, n = 16 patients with MDR-TB; Period 2, n = 22 patients with MDR-TB
										• MDR-TB risk was 4.8% (4/84) for those with exposures to periods 1 and 2; and 0.5% (4/863) for AIDS patients without same-ward exposure
Wenger, 1995 [48]	USA	Yes	Yes	Yes	Yes	TST conversion | HCWs	Jan–May 1990: 28% (7/25)	Jun 1990–Feb 1991 [early]: 18% (3/17);	χ ^2^ for trend (3 time-periods), *P* < .01	• Stringent isolation criteria were only put into effect in Feb 1991
								Mar 1991–Jun 1992 [late]: 0% (0/23)		
Bryan, 1983 [49]	USA	No	Yes	No	Yes	TST conversion | HCWs	1976: 4.5%	1977: 5.1%;		• Possible problem of faulty performance of test/ presence of booster phenomenon in 1976–1977
								1979: 1.5%;		
								1980: 0.85%;		
								1981: 0.59%		
										• n/N not reported
										• Fidelity of intervention (proportion of patients with culture confirmed TB who were isolated): 1976: 3/15; 1977: 9/24; 1978: 8/23; 1979: 18/30; 1980: 14/26
Jacobson, 1957 [21]	USA	Yes	No	No	No	TB disease incidence rate | HCWs	1942–1951 (overall time period): 2.0/1,000 PY (78 events in 38 331 PY);	1952–1953: 1.0/1,000 PY (9 events in 9,030 PY); 1954–1955: 0.3/1000 PY (3 events in 9,199 PY)	Not reported.	• Peak in 1948–1950 coincided with community wide case-finding activities
										• Also showed data by HCW occupation

Abbreviations: adj., adjusted; AFB, acid-fast bacilli; CDC, Centers for Disease Control and Prevention; CI, confidence interval; HCWs, healthcare worker; Isol^n^, isolation or spatial separation; IPC, infection prevention and control; LTBI, latent TB infection; MDR, multidrug-resistant; misc., miscellaneous; mth, month; OR, odds ratio; Oth, other; PD, person-days; PM, person-months; PPE, personal protective equipment; pt, patient; PY, person-years; ref, reference; SD, standard deviation; SI/ID, Special Immunology/Infectious Disease; TB, tuberculosis; Tri^g^, triage of people with signs or symptoms of TB; TST, tuberculin skin test; Tx, effective treatment based on drug susceptibility; unadj., unadjusted; unk., unknown; Y, yes.

^a^Based on WHO 2016 definitions [15].

^b^Includes administrative, personal protective, and environmental IPC measures.

^c^Rate ratios derived from data presented and not included in authors’ analysis.

^d^Conference abstract only.

Six studies reported changes in TB disease incidence; 1 that used only triage reported 78 episodes in 38 331 people-years (PY) before implementation versus 12 episodes in 18 229 PY after implementation (calculated IR ratio 0.32) [21]. Two other studies in low burden settings also showed reduced risk/incidence after implementation of composite interventions [34, 47]. In contrast, 3 studies in high burden settings [24, 28, 29] showed small or no reductions in risk/incidence after use of triage (and other interventions), from 3.7% to 3.2% [29], 0.65 to 0.44 per 100 PY [28], and an adjusted odds ratio (OR) of 0.97 (95% confidence interval [CI], .90–1.04) [24] comparing hospitals with higher versus lower administrative scores (Table 4).

### Studies Implementing Isolation or Spatial Separation

Twenty-four studies [22, 24–29, 31–38, 40–43, 45–49] implemented respiratory isolation or spatial separation; 18/24 (75%) were in low burden countries. All studies, except 1 [22], used isolation together with other TB IPC measures.

Among the 19 studies [22, 26–28, 31–33, 35–38, 40–43, 45, 46, 48, 49] reporting differences in LTBI incidence, effects ranged from a 1% increase (n = 4060) [33] to a 20.5% reduction (n = 65; Table 4) [48]. The 2 largest studies (one each in the USA and Brazil) showed absolute reductions in LTBI incidence (1.2% [42] and 1.7%) [27]. Among 6 studies reporting IRs, IR ratios (intervention vs no intervention) ranged from 0.01 (95% CI, 0–.04; *P* < .001; adjusted, covariates unclear) [28] to 0.24 (95% CI, .10–.54; adjusted for exposure and occupation) [26] and 0.46 (calculated from data) [38]. Among the 6 studies that reported changes in TB disease, estimates of effect differed by setting, from almost no difference in incidence in the 4 studies in high burden settings [24, 25, 28, 29], to absolute reductions of 6% (n = 409) [47] and 29% (n = 134; calculated) [34] in low burden settings.

### Studies Implementing Effective Treatment Based on Drug Susceptibility

Five studies [31, 45–48] used effective treatment with other IPC measures. “Effective treatment” was defined variably, from a change in regimen from 3 to 4 drugs [48], to the use of “radiometric susceptibility testing,” [31] which, it was assumed, would have led to appropriate treatment, though this is not stated.

Two studies did not report outcomes for all participants/sites [31, 45]. All studies showed absolute reductions in TST conversion after implementation of IPC measures, ranging from 2.1% [46] to 20.5% [48] (crude; calculated), although all studies had small numbers of outcomes (range 10–104) and 2 had small sample sizes (n ≤ 650).

Only 1 study [47] used effective treatment and measured TB disease incidence, employing an “expanded anti-TB regimen” (a change from median 1.5 [range 0–4] to 2.0 [range 0–4] drugs) as part of a composite intervention that included triage, isolation, and changes to diagnostic processes. They found a change in TB disease risk (or “attack rate”) among HIV-positive individuals admitted to the ward, from 8.8% before, to 2.6% after intervention (*P* = .01).

### Quality Assessment and GRADE

All 18 retrospective studies scored poorly (median 10/27 [interquartile range 8.3–12.0; range 6–13]; Table 5). The 7 prospective studies also scored poorly (Table 6): 1 study was marked down for incomplete outcome reporting and 3 for selective outcome reporting. The overall low study quality was reflected in the GRADE assessment (Appendix 3; Supplementary Tables 4–9), where the strength of evidence was consistently downgraded due to serious risk of bias and very serious indirectness (the latter often due to the concurrent use of multiple IPC measures).

**Table 5.** Summary of Quality Assessments for Retrospective Studies^a^ (n = 18 Studies)

First Author	Year	Reporting (max = 11)	External Validity (max = 3)	Internal Validity: Bias (max = 7)	Internal Validity: Confounding (max = 6)	Total, n (%/27)
Bangsberg	1999	5	2	4	1	12 (44.4)
Blumberg	1995	6	2	4	1	13 (48.2)
Blumberg	1997	6	2	4	1	13 (48.2)
Bryan	1983	3	1	1	1	6 (22.2)
Claassens	2013	6	1	2	2	11 (40.7)
Fridkin	1995	4	0	2	1	7 (25.9)
Harries	2002	7	1	4	1	13 (48.1)
Holzman	1995	4	2	2	2	10 (37.0)
Jacobson	1957	4	0	3	2	9 (33.3)
Jarvis	1995	6	1	4	2	13 (48.1)
Jones	2002	3	1	2	2	8 (29.6)
Louther	1997	3	2	2	2	9 (33.3)
Maloney	1995	5	2	2	1	10 (37.0)
O’Hara	2017	7	1	1	3	12 (44.4)
Sinkowitz	1996	6	0	4	2	12 (44.4)
Stroud	1995	4	0	2	1	7 (25.9)
Uyamadu	1997	3	0	2	1	6 (22.2)
Welbel	2009	5	0	3	1	9 (33.3)

^a^Assessed using the Downs & Black tool [16].

**Table 6.** Summary of Quality Assessment for Prospective Studies^a^ (n = 7 Studies)

First Author	Year	Sequence Generation	Allocation Concealment	Blinding	Incomplete Outcome data	Selective Outcome Reporting	Other Sources of Bias
Baussano	2007	No	No	No	Yes	No	No
Behrman	1998	No	No	No	No	No	No
da Costa	2009	No	No	No	No	No	No
Moro	2000	No	No	No	No	Yes	No
Roth	2005	No	No	No	No	Yes	No
Wenger	1995	No	No	No	No	No	No
Yanai	2003	No	No	No	No	Yes	No

^a^Assessed using the Cochrane collaboration tool for experimental studies and prospective cohort studies (http://www.cochrane-handbook.org)

## DISCUSSION

This review found 25 studies, published from 1957 to 2017, that reported the effects of triage, isolation, and effective treatment on the incidence of LTBI or TB disease in HCWs and others attending health facilities. Most studies were conducted in the 1990s in US hospitals, several in response to outbreaks of TB [22, 34, 45–48]. Almost all studies showed reduced LTBI or TB disease incidence after implementation of a package of IPC measures, but because of heterogeneity in study design and reporting of results, meta-analysis was not conducted. Studies were generally of low quality. All studies, except 2, tested composite interventions, including other administrative measures, PPE, and environmental measures; it was therefore not possible to disaggregate the effects of specific interventions from those of the others described.

It is important that these findings should not be interpreted to suggest a lack of efficacy of the TB IPC measures examined; effective treatment, in particular, is supported by studies outside health care settings [50] and studies from healthcare settings using guinea pigs as infection endpoints [13]. Indeed, the WHO 2019 TB IPC guidelines recommend all 3 examined measures as first-line controls to be used as part of broad suite of interventions [12]. Statements, in the guidelines, around “low certainty” and “indirectness” reflect the overall poor study quality, heterogeneity in study design, implementation of multiple interventions at 1 time, predominance of studies from a particular type of setting, and deficiencies in the reporting of results. These issues are discussed below.

### Gaps in the Literature

Most studies were from high-income, low TB burden settings, predominantly the United States. Conspicuously absent were countries with very high TB burdens, such as India and China, and countries in sub-Saharan Africa and South or Central America (other than South Africa, Malawi, and Brazil), where the LTBI burden among HCWs is known to be very high [4, 7]. Data from these countries are essential if global policy is to address successfully the broad range of environments in which IPC measures must be implemented.

Only 1 study was conducted in a primary care setting [25]. Although many people with TB in low burden countries may receive treatment in hospitals, most in high burden countries are cared for as out-patients and may not visit a hospital at any point in their illness [51]. WHO widely recommends the decentralization of TB care [52, 53], although for DR-TB this policy is variably effected [2]. As shown in South Africa [54–57], HCWs in clinics and the community are also at high risk of TB infection and disease. Evidence is still needed for the effectiveness of IPC measures in these environments, which present different challenges for implementing interventions and measuring outcomes [58, 59].

Many studies provided detailed descriptions of interventions used, but often did not describe, in any depth, fidelity to these interventions. Cross-sectional studies [24, 25, 40, 42] were the weakest in this regard, as they were able only to assess whether an intervention or policy had been instated and not if it was being applied as intended. (Additional methodological shortcomings in some cross-sectional studies further reduced confidence in their findings; for example, the study by Claassens et al [25], where IPC coverage was estimated after the period during which outcomes were enumerated.) Some reporting of fidelity is essential to strengthen what is already very indirect evidence for the effectiveness of these interventions.

A consistent finding was the lack of reporting of secular changes in TB incidence or prevalence among people attending the facility over the course of the study. This is particularly relevant given the high number of before-after or during-after studies included, where the same facility/ies at different time points served as control and intervention. Changes in the numbers of potentially infectious individuals attending study facility/ies may have had a dramatic effect on the risk of MTB transmission to HCWs, and reductions in LTBI or active TB incidence may have been misattributed to the implementation of IPC measures. Measurement of secular changes is recommended by guidance on conducting before-after studies [19] and should be a standard reporting requirement for future studies.

Incidence of LTBI or TB disease in HCWs are useful ways to estimate MTB transmission from patients in health care settings. Transmission between patients and from HCWs to patients does, of course, occur, although this was measured by only 2 studies, both in low TB burden, high-income countries [34, 47]. Choice of at-risk population, outcomes, and outcome measurement are critical when studying MTB transmission, but can also make study design more complex [60]. In high TB burden countries, a high proportion of HCWs already have LTBI, limiting the size of the at-risk population. Using TST to measure LTBI incidence (as in several of the included studies) can also be problematic, as reactions can vary based on host factors. The development of TB disease, though easier to measure, is also dependent on a number of interconnected host factors and, in the absence of complementary molecular epidemiological data, is more difficult to reliably attribute to a congregate setting transmission event. More detailed descriptions of at-risk HCW populations would allow for better extrapolation of findings to other key populations, particularly HIV-positive individuals, and provide better guidance on how to prevent TB in HCWs. As discussed by Harries et al [29], robust occupational health programs are critical to the well-being of frontline HCWs; embedding TB IPC studies within existing occupational health frameworks may allow for better reporting of individual HCW risk profiles and improve long-term fidelity to interventions.

### Future Research

This review, like others [4, 6, 7, 11, 61], found limited and low quality evidence for the effectiveness of administrative IPC measures in reducing MTB transmission, with overrepresentation of data from hospitals in high-income, low TB burden countries. Like previous reviewers, we call for better designed and implemented studies from a wider variety of settings, although we acknowledge the difficulties of doing this in what are often unpredictable environments, and recognize the shortcomings in the methods available to measure MTB transmission in these settings [62, 63].

Despite the weaknesses in the data presented here, the weight of evidence to support the use of established TB IPC measures is sufficient that it would be unethical to conduct randomized trials involving a true “control” arm, although trials comparing “best practice” IPC interventions with an established basic standard of care should still be considered, as should the use of pragmatic trial designs, such as stepped wedge cluster randomized trials [64]. We would suggest a change in expectations and an acceptance of the limitations inherent in conducting these complex interventional studies in challenging clinical settings. Standardization of study designs, outcome measurement, and reporting formats, with replication of clusters or sites would facilitate the generation of more robust data syntheses to guide policy making, as would efforts by investigators to provide more precise and comprehensive data in the areas discussed above. We suggest that greater numbers of imperfect but comparable data from studies conducted in a wide range of settings that adhere to a set of standardized rules around design, and reporting would be more useful to decision making than a few perfectly designed studies conducted in places unrepresentative of those where effective interventions are most needed. Additionally, quasi-experimental techniques, such as interrupted time-series analysis [65–67], with or without controls [68], or difference of differences approaches have been employed with success in evaluating complex public health policy interventions in rapidly changing environments [69], and should be considered seriously for future real-world estimations of the effectiveness of TB IPC measures. To this end, given the difficulties outlined above around measurement of outcomes, confounding, bundling of interventions, and valid comparator groups, it would be beneficial to have additional specific guidance, developed by relevant experts, to help investigators plan, conduct, and report studies examining the efficacy of measures to reduce MTB transmission.

### Limitations and Strengths

“Prompt initiation of effective treatment” is widely considered a reliable way to reduce MTB transmission, and is the wording used in the WHO 2019 TB IPC guidelines [12]. The 5 studies included in this review that used effective treatment did not report time to treatment, and because “prompt initiation of effective treatment” was not one of the defined interventions of interest, studies examining its efficacy in reducing transmission were not included for analysis.

Heterogeneity of the data and weaknesses in study design prevented meaningful quantitative synthesis, which may have provided a clearer guide for policy makers. Studies may have been overlooked during sifting or published in nonspecified languages. Strengths include the application of a robust search strategy by a professional librarian across a wide range of repositories, all sifting and data extraction being done in duplicate (per PRISMA recommendations; Appendix 4) [70], and the use of GRADE to assess quality.

## CONCLUSIONS

This review found 25 studies implementing triage, isolation, or effective treatment, and measuring the incidence of LTBI or TB disease or both. Overall, packages of IPC measures appeared to reduce MTB transmission, but studies were of low quality and evidence for the effectiveness of individual or combined measures was indirect and of limited utility; heterogeneity of the data prevented meta-analysis. More data are needed from high-burden, lower-income, primary care settings. Harmonization of study designs and reporting frameworks will allow for more formal data syntheses, creating a better platform for policy making. The development of specific guidance around conducting and reporting studies to determine the efficacy of TB IPC measures should be prioritized by governing and stakeholder bodies.

## Supplementary Data

Supplementary materials are available at *Clinical Infectious Diseases* online. Consisting of data provided by the authors to benefit the reader, the posted materials are not copyedited and are the sole responsibility of the authors, so questions or comments should be addressed to the corresponding author.

ciaa720_suppl_Supplementary-Material

## Notes

***Author contributions.*** Conceptualization: A. S. K., D. A. J. M., J. F., K. L. F., R. C. H.

Data curation: A. S. K., K. L. F., R. C. H.

Formal analysis: A. S. K., K. L. F., M. G.

Funding acquisition: D. A. J. M., K. L. F.

Investigation: A S. K., D. A. J. M., H. E. B., I. G, J. E., J. F., K. L. F., M. C., M. G., M. T., R. C. H.

Methodology: A. S. K., D. A. J. M., J. F., K. L. F., M. G., R. C. H.

Project administration: A. S. K., D. A. J. M., K. L. F., R. C. H.

Resources: J. F., M. G., R. C. H.

Software: n/a

Supervision: D. A. J. M., K. L. F., R. C. H.

Visualization: A. S. K.

Writing—original draft: A. S. K., K. L. F.

Writing—review and editing: A. S. K., D. A. J. M., H. E. B., I. G., J. E., J. F., K. L. F., M. C., M. G., M. T., R. C. H,

***Acknowledgments.*** Thanks to Lice González-Angulo, Fuad Mirzayev, Thomas Piggott, and Holger Schünemann for their guidance and help with GRADE, in particular.

***Financial support.*** This work was supported by the World Health Organization (reference 2017/748478-0) and the Medical Research Council, UK (reference MR/K012126/1). The funders had no role in study design, data collection and analysis, decision to publish, or preparation of the manuscript.

***Potential conflicts of interest.*** A. K. reports holding a part-time contract at the Institute for Global Health and Development, Queen Margaret University, Edinburgh; consulting fees from The Aurum Institute, South Africa; grants through LSHTM from Economic and Social Research Council, UK; grants through QMU (grant awarded to University College London) from National Institute of Health Research, UK; grants through LSHTM from The Bloomsbury SET (Research England); and consulting fees from Edanz Group Japan Ltd., outside the submitted work. I. G. reports grants awarded to LSHTM from Economic and Social Research Council, UK and National Institutes of Health, outside of submitted work. R. H. reports personal fees from Sanofi Pasteur, outside the submitted work. All other authors report no potential conflicts. All authors have submitted the ICMJE Form for Disclosure of Potential Conflicts of Interest. Conflicts that the editors consider relevant to the content of the manuscript have been disclosed.
