# Supplementary material for: Evidence for the Use of Triage, Respiratory Isolation, and Effective Treatment to Reduce the Transmission of Mycobacterium Tuberculosis in Healthcare Settings: A Systematic Review
Source: Clin Infect Dis. 2020 Jun 5;72(1):155–72. doi: 10.1093/cid/ciaa720 (PMC7823078; doi:10.1093/cid/ciaa720)
Supplement: ciaa720_suppl_Supplementary-Material [file ciaa720_suppl_supplementary-material.docx]

Evidence for the use of administrative controls to reduce the transmission of *Mycobacterium tuberculosis* in health care settings: a systematic review

Supplementary material

Table of Contents

[Appendix 1: Additional methods 3](#_Toc30865373)

[Population, interventions, comparators, and outcomes 3](#_Toc30865374)

[Details of bibliographic search 3](#_Toc30865375)

[Search strategy, terms, and sources 3](#_Toc30865376)

[Selection of studies and inclusion and exclusion criteria 7](#_Toc30865377)

[Data extraction and management 7](#_Toc30865378)

[Appendix 2: Additional results 8](#_Toc30865379)

[Appendix 3: GRADE assessment 9](#_Toc30865380)

[Question 1: Can triage of people with TB signs and/or symptoms be used in health care settings to reduce TB transmission to HCWs (including CHWs) when compared to transmission to HCWs (including) CHWs in settings with no or different interventions? 9](#_Toc30865381)

[Question 1: Explanations 10](#_Toc30865382)

[Question 1: References 13](#_Toc30865383)

[Question 2: Can respiratory isolation (spatial separation) of presumed or demonstrated infectious TB cases reduce TB transmission to HCWs (including CHWs) when compared to transmission to HCWs (including CHWs) in settings with no intervention or different interventions? 15](#_Toc30865384)

[Question 2: Explanations 16](#_Toc30865385)

[Question 2: References 19](#_Toc30865386)

[Question 3: Can effective treatment of TB disease reduce TB transmission to HCWs (including CHWs) when compared to transmission to the same populations in settings where treatment is not yet administered? 21](#_Toc30865387)

[Question 3: Explanations 22](#_Toc30865388)

[Question 3: References 22](#_Toc30865389)

[Question 4: Can triage of people with TB signs, symptoms reduce TB transmission to other persons attending healthcare settings when compared to transmission to the same populations in settings with no intervention or different interventions? 23](#_Toc30865390)

[Question 4: Explanations 24](#_Toc30865391)

[Question 4: References 24](#_Toc30865392)

[Question 5: Can respiratory isolation (spatial separation) reduce TB transmission to other persons attending healthcare settings when compared to transmission to the same populations in settings with no intervention or different interventions? 25](#_Toc30865393)

[Question 5: Explanations 26](#_Toc30865394)

[Question 5: References 26](#_Toc30865395)

[Question 6: Can effective treatment of TB disease reduce TB transmission to other persons attending healthcare settings when compared to transmission to the same populations in settings where treatment administration is delayed? 27](#_Toc30865396)

[Question 6: Explanations 28](#_Toc30865397)

[Question 6: References 28](#_Toc30865398)

[Appendix 4: PRISMA* checklist 29](#_Toc30865399)

[Appendix 5: Additional references 32](#_Toc30865400)

# Appendix 1: Additional methods

## Population, interventions, comparators, and outcomes

The populations of interest were: (1) HCWs (including community health workers [CHWs]) working in healthcare settings with applied intervention/s; and (2) non-HCWs (other staff working in a healthcare setting, patients, and visitors) attending healthcare settings with applied intervention/s. The interventions of interest were any of the following, implemented in a health care setting either individually or in any combination: (1) triage of people with signs or symptoms of TB or confirmed TB; (2) respiratory isolation (including spatial separation) of presumed infectious TB cases (the basis for isolation could include microbiological, radiological, or clinical criteria); and (3) effective treatment of TB based on bacteriologic susceptibility. Comparator groups used were: (1) HCWs (including CHWs) working in healthcare settings with no intervention or different intervention; and (2) non-HCWs (other staff working in a healthcare setting, patients, and visitors) attending healthcare settings with no intervention or different intervention. Outcomes of interest were: (1) difference in LTBI incidence/prevalence; (2) difference in TB disease incidence/prevalence; or (3) incidence/prevalence ratio of LTBI or TB disease (or other measures of relative difference).

## Details of bibliographic search

### Search strategy, terms, and sources

Lists of search terms were circulated to all authors for discussion before the construction of the search strategy. The search strategy was compiled and tested on the OvidSP Medline database before it was edited and run across other relevant information sources. Search strategies were checked by a professional librarian not connected with the research team for typographical or syntax errors. The search strategies used subject headings, where available, and search terms were run in the title and abstract, where possible. Due to language skills available in the research team, literature was limited to papers written in English, Japanese, Chinese, Russian, French, Spanish, or Portuguese. Animal studies were excluded. No date limits or publication type limits were added to the search.

A search was constructed in OvidSP Medline using variations and sub-categories of the following terms: “tuberculosis”, “administrative infection control”, “bacteriologic susceptibility tests”, “triage”, “respiratory isolation”, and “disease transmission” (see Supplementary table 1 for detailed search strategy). This was adapted, as appropriate, for the other search sources, which included Web of Science databases, Scopus®, the Cochrane library, the Cumulative Index to Nursing and Allied Health Literature (CINAHL) databases, and grey literature databases (see Supplementary table 2 for details of databases searched). The final search was run on 30 November 2017.

Supplementary table 1. Detailed search strategy implemented in OVIDSP Medline and modified for other databases

| # | Term |
| --- | --- |
| 1 | exp tuberculosis/ |
| 2 | Mycobacterium tuberculosis/ |
| 3 | tuberculosis.ti,ab. |
| 4 | tuberculin.ti,ab. |
| 5 | tb.ti,ab. |
| **6** | **or/1-5 [TUBERCULOSIS]** |
| 7 | administrat* control.ti,ab. |
| 8 | (administrat* adj3 (transmis* control or infect* control)).ti,ab. |
| **9** | **or/7-8 [ADMINISTRATIVE INFECTION CONTROL]** |
| 10 | bacteri* suscept*.ti,ab. |
| 11 | suscept* test*.ti,ab. |
| 12 | sensitiv* test*.ti,ab. |
| 13 | ADST.ti,ab. |
| 14 | molecul* screen*.ti,ab. |
| 15 | GeneXpert.ti,ab. |
| 16 | xpert.ti,ab. |
| 17 | line probe assay.ti,ab. |
| 18 | molecular assay.ti,ab. |
| 19 | nucleic acid amplification test*.ti,ab. |
| 20 | (NAAT or NAATs).ti,ab. |
| 21 | MTBDRplus.ti,ab. |
| 22 | rifampicin resistan*.ti,ab. |
| 23 | isoniazid resistan*.ti,ab. |
| **24** | **or/10-23** |
| 25 | infect*.ti,ab. |
| 26 | transmis*.ti,ab. |
| 27 | transmit*.ti,ab. |
| 28 | expos*.ti,ab. |
| 29 | (acquisition* or acquire*).ti,ab. |
| 30 | risk*.ti,ab. |
| **31** | **or/25-30** |
| **32** | **24 and 31 [BACTERIOLOGIC SUSCEPTIBILITY TESTS]** |
| 33 | Triage/ |
| 34 | triage.ti,ab. |
| 35 | ((transfer or distribution or referral or referred or assessment or assess) adj2 (severity or priority or prioriti#e)).ti,ab. |
| 36 | assign priority.ti,ab. |
| 37 | (systematic adj2 admission).ti,ab. |
| 38 | (patient adj3 (evaluat* or sorting or sort)).ti,ab. |
| **39** | **or/33-38 [TRIAGE]** |
| 40 | Patient Isolation/ |
| 41 | respirat* isolat*.ti,ab. |
| 42 | ((patient* or inpatient* or in-patient* or outpatient* or out-patient*) adj2 isolat*).ti,ab. |
| 43 | air* infect* isolat*.ti,ab. |
| 44 | AII.ti,ab. |
| 45 | air* isolat*.ti,ab. |
| 46 | negative-pressure isolat*.ti,ab. |
| 47 | negative-pressure room*.ti,ab. |
| 48 | spatial separat*.ti,ab. |
| 49 | distancing.ti,ab. |
| **50** | **or/40-49 [RESPIRATORY ISOLATION]** |
| **51** | **9 or 32 or 39 or 50 [ALL INTERVENTIONS]** |
| **52** | **6 and 51** |
| 53 | infectious disease transmission, patient-to-professional/ |
| 54 | infectious disease transmission, professional-to-patient/ |
| **55** | **or/53-54 [DISEASE TRANSMISSION BETWEEN HCWs AND PATIENT]** |
| **56** | **6 and 55** |
| **57** | **52 or 56** |
| 58 | Humans/ |
| 59 | Animals/ |
| **60** | **58 and 59** |
| **61** | **59 not 60 [ANIMAL STUDIES ONLY]** |
| **62** | **57 not 61** |
| 63 | (chinese or english or french or japanese or portuguese or russian or spanish).lg. |
| **64** | **62 and 63** |
| **65** | **remove duplicates from 64** |

Terms ending in “/” are MeSH terms. Terms ending in “ti,ab” limit the search to the title and abstract only. Terms ending in “lg” limit the search to language only. Words enclosed in square brackets are ignored by the search. “ADJn” searches for terms within n words of each other. “*” is the truncation symbol. “#” is the wildcard symbol.
The full search strategy can also be accessed on the London School of Hygiene & Tropical Medicine data compass^1^

Supplementary table 2. Databases searched

| Database/source | | Time period | Date searched |
| --- | --- | --- | --- |
| **Bibliographic databases** | |  |  |
| 1 | OvidSP MEDLINE In-Process & Other Non-Indexed Citations, Ovid MEDLINE Daily and Ovid MEDLINE | 1946–present | 09 November 2017 |
| 2 | OvidSP EMBASE Classic + Embase | 1947–2017 (08 November) | 09 November 2017 |
| 3 | EBSCO CINAHL Plus | Complete database | 10 November 2017 |
| 4 | OvidSP Global Health 1910 to present | 1910–2017 (Week 43) | 09 November 2017 |
| 5 | Elsevier SCOPUS | Complete database | 10 November 2017 |
| 6 | WoS Core Collection (Citation Index Expanded (SCI-EXPANDED) | 1970–2017 (09 November) | 10 November 2017 |
| 7 | WoS Social Sciences Citation Index (SSCI) | 1970–2017 (09 November) | 10 November 2017 |
| 8 | WoS Arts & Humanities Citation Index (A&HCI) | 1975–2017 (09 November) | 10 November 2017 |
| 9 | WoS Emerging Sources Citation Index (ESCI) | 2015–2017 (09 November) | 10 November 2017 |
| 10 | EBSCO Africa-Wide Information | Complete database | 11 November 2017 |
| 11 | WoS Korean Journals Database | 1980-2017 (02 November) | 10 November 2017 |
| 12 | WoS Russian Science Citation Index | 2005–2017 (09 November) | 10 November 2017 |
| 13 | WoS SciELO Citation Index | 1997–2017 (09 November) | 10 November 2017 |
| 14 | Wiley Cochrane Library (Cochrane Database of Systematic Reviews; DARE; Cochrane Central Register of Controlled Trials; Health Technology Assessments; Economics Evaluations) | Complete database. CDSR 2017, Issue 11 | 10 November 22017 |
| 15 | Bireme Virtual Health Library LILACS | Complete database | 10 November 2017 |
| **Grey literature** | |  |  |
| 1 | Open Grey | Complete database | 10 November 2017 |
| 2 | OvidSP Northern Light Life Sciences Conference Abstracts | 2010–2017 (Week 43) | 09 November 2017 |
| 3 | Conference Proceedings Citation Index- Science (CPCI-S) | 1990–2017 (09 November) | 10 November 2017 |
| 4 | Conference Proceedings Citation Index- Social Science & Humanities (CPCI-SSH; searched as part of the WoS Core Collection). | 1990–2017 (09 November) | 10 November 2017 |
| 5 | New York Academy of Medicine Grey Literature Report |  |  |
| 6 | WHO ICTRP | Complete database | 10 November 2017 |
| 7 | ClinicalTrials.gov | Complete database | 10 November 2017 |
| 8 | International Union against Tuberculosis and Lung Disease Conference Electronic Abstract Database | 2017 only |  |

EBSCO: Elton B. Stephens Co.; ICTRP: International Clinical Trials Registry Platform; CINAHL: Cumulative Index to Nursing and Allied Health Literature; DARE:Database of Abstracts of Reviews of Effects; EMBASE: Excerpta Medica DataBASE; LILACS: Literatura Latino-Americana em Ciências da Saúde; MEDLINE: Medical Literature Analysis and Retrieval System Online; SciELO: Scientific Electronic Library Online; WHO: World Health Organization; WoS: Web of Science

## Selection of studies and inclusion and exclusion criteria

Sifting and data extraction were conducted in duplicate by two reviewers. A two-stage sifting process was employed to screen publications at: (1) title and abstract; and (2) full text level for eligibility for inclusion using the criteria in Table 1 of the main article. A check of all inclusions after title and abstract sifting was conducted by a third reviewer; if additional articles were identified for exclusion, a majority decision was reached through discussion with the original reviewers or independent reviewers. Additional titles identified from the reference lists and citations of all included articles were sifted in the same way and included if eligible. Citation tracking was conducted in Web of Science and/or Scopus®. Any unresolved disagreements in sifting were resolved by a third, independent reviewer.

## Data extraction and management

Lists of articles, including reasons for inclusion and exclusion, were maintained in Microsoft® (MS) Excel spreadsheets; articles were organised using Mendeley or Endnote®. Data were extracted by two independent reviewers into separate piloted and standardised MS Excel spreadsheets and a single consensus dataset produced after discussion, as necessary. In cases where consensus could not be reached, the article and data were reviewed by a third, independent reviewer and a decision reached after discussion between reviewers. Data extracted included HIV status and age of individuals in the population; population role (e.g., HCW, patient, visitor); healthcare setting (e.g., TB clinic, TB ward, MDR-TB ward); high or low TB burden setting (based on 2016 WHO definitions);^2^ details of intervention/s implemented; details of method of measuring outcome/s; numbers of outcomes in intervention and control groups; and crude and adjusted measures of effect, including details of any covariates used to make adjustment.

# Appendix 2: Additional results

Supplementary table 3. List of systematic reviews (n = 19) and guidelines (n = 6) for which reference lists were reviewed, listed in reverse chronological order of publication

| **First author [ref]** | **Year published** | **Title** |
| --- | --- | --- |
| Schmidt* ^3^ | 2018 | Effectiveness of control measures to prevent occupational tuberculosis infection in health care workers: a systematic review |
| Nathavitharana ^4^ | 2017 | Agents of change: The role of healthcare workers in the prevention of nosocomial and occupational tuberculosis |
| Nasreen ^5^ | 2016 | Prevalence of latent tuberculosis among health care workers in high burden countries: a systematic review and meta-analysis |
| Punjabi ^6^ | 2016 | Preventing transmission of *Mycobacterium tuberculosis* in health care settings |
| van Cutsem ^7^ | 2016 | Infection control for drug-resistant tuberculosis: early diagnosis and treatment is the key |
| Verkuijl ^8^ | 2016 | Protecting our front-liners: occupational tuberculosis prevention through infection control strategies |
| Zuckerman ^9^ | 2011 | Prevention of health care-acquired pneumonia and transmission of *Mycobacterium tuberculosis* in health care settings |
| Trajman ^10^ | 2010 | Occupational respiratory infections |
| Conde ^11^ | 2009 | III Brazilian thoracic association guidelines on tuberculosis |
| WHO ^12^ | 2009 | WHO policy on TB infection control in health-care facilities, congregate settings and households |
| Humphreys ^13^ | 2007 | Control and prevention of healthcare-associated tuberculosis: the role of respiratory isolation and personal respiratory protection |
| Menzies ^14^ | 2007 | Risk of tuberculosis infection and disease associated with work in health care settings |
| Joshi ^15^ | 2006 | Tuberculosis among health-care workers in low- and middle-income countries: a systematic review |
| Tam ^16^ | 2006 | Occupational tuberculosis: a review of the literature and the local situation |
| Jensen (USA CDC) ^17^ | 2005 | Guidelines for preventing the transmission of *Mycobacterium tuberculosis* in health-care settings, 2005 |
| Sepkowitz ^18^ | 2001 | Tuberculosis control in the 21st century |
| Raymond ^19^ | 1998 | ACOEM guidelines for protecting health care workers against tuberculosis |
| Cookson ^20^ | 1997 | Prevention of nosocomial transmission of *Mycobacterium tuberculosis* |
| Davis ^21^ | 1997 | Hospital infection control practices for tuberculosis |
| Harries ^22^ | 1997 | Practical and affordable measures for the protection of health care workers from tuberculosis in low-income countries |
| Canada CDC ^23^ | 1996 | Guidelines for preventing the transmission of tuberculosis in Canadian health care facilities and other institutional settings |
| L’Ecuyer ^24^ | 1996 | Further progress in the protection of healthcare workers |
| McGowan ^25^ | 1995 | Nosocomial tuberculosis: new progress in control and prevention |
| Menzies ^26^ | 1995 | Tuberculosis among health care workers |
| Seyferth ^27^ | 1994 | Guidelines for preventing occupational exposure to TB |

*Published after literature search was completed
ACOEM: American College of Occupational and Environmental Medicine; CDC: Centers for Disease Control and Prevention; ref: reference; TB: tuberculosis; USA: United States of America; WHO: World Health Organization

# Appendix 3: GRADE assessment

## Question 1: Can triage of people with TB signs and/or symptoms be used in health care settings to reduce TB transmission to HCWs (including CHWs) when compared to transmission to HCWs (including) CHWs in settings with no or different interventions?

Supplementary table 4. Question 1: Evidence summary

| **Certainty assessment** | | | | | | | **№ of patients** | | **Effect** | | **Certainty** | **Importance** |
| --- | --- | --- | --- | --- | --- | --- | --- | --- | --- | --- | --- | --- |
| **№ of studies** | **Study design** | **Risk of bias** | **Inconsist-ency** | **Indirectness** | **Imprecision** | **Other con-siderations** | **Triage** | **No triage** | **Relative (95% CI)** | **Absolute (95% CI)** |  |  |
| **Reduction in LTBI incidence/prevalence in all settings ^a^** | | | | | | | | | | | | |
| 6 ^1,2,3,4,5,6,b,c,d,e,f^ | observational studies ^g^ | serious ^h^ | not serious | very serious ^i^ | serious ^j^ | none | 1966/24852 (7.9%) | 1350/9647 (14.0%) | **RR 0.57** (-- to --) | **60 fewer per 1,000** (from -- to --) | ⨁◯◯◯ VERY LOW | CRITICAL |
| **Reduction in LTBI incidence/prevalence in low TB burden settings ^k^** | | | | | | | | | | | | |
| 5 ^2,3,4,5,6,b,c,f,l^ | observational studies ^g^ | serious ^h^ | not serious | very serious ^i^ | serious ^m^ | none | 206/22035 (0.9%) | 322/8045 (4.0%) | **RR 0.23** (-- to --) | **31 fewer per 1,000** (from -- to --) | ⨁◯◯◯ VERY LOW | CRITICAL |
| **Reduction in LTBI incidence/prevalence in high TB burden settings ^n^** | | | | | | | | | | | | |
| 1 ^1,d^ | observational studies | serious ^o^ | not serious ^p^ | serious ^q^ | not serious | none | 1760/2817 (62.5%) | 1028/1602 (64.2%) | **RR 0.97** (-- to --) | **19 fewer per 1,000** (from -- to --) | ⨁◯◯◯ VERY LOW | CRITICAL |
| **Reduction in LTBI incidence/prevalence in primary care - not measured** | | | | | | | | | | | | |
| - | - | - | - | - | - | - |  | | | | - |  |
| **Reduction in LTBI incidence/prevalence in secondary/tertiary care ^r^** | | | | | | | | | | | | |
| 6 ^1,2,3,4,5,6,b,c,d,e,f^ | observational studies ^g^ | serious ^h^ | not serious | very serious ^i^ | serious ^j^ | none | 1966/24852 (7.9%) | 1350/9647 (14.0%) | **RR 0.57** (-- to --) | **60 fewer per 1,000** (from -- to --) | ⨁◯◯◯ VERY LOW | CRITICAL |
| **Reduction in active TB incidence/prevalence in all settings ^s^** | | | | | | | | | | | | |
| 2 ^7,8,t,u,v^ | observational studies | serious ^w^ | not serious | very serious ^x^ | serious ^y^ | none | 110/6216 (1.8%) | 129/7161 (1.8%) | **RR 0.98** (-- to --) | **0 fewer per 1,000** (from -- to --) | ⨁◯◯◯ VERY LOW | CRITICAL |
| **Reduction in active TB incidence/prevalence in low TB burden settings** | | | | | | | | | | | | |
| 1 ^9^ | observational studies | not serious | not serious ^p^ | not serious | serious ^z^ | none |  |  | **RR 0.32** (-- to --) | **0 fewer per 1,000** (from -- to --) | ⨁◯◯◯ VERY LOW | CRITICAL |
| **Reduction in active TB incidence/prevalence in high TB burden settings ^aa^** | | | | | | | | | | | | |
| 2 ^7,8,u,v^ | observational studies | serious ^w^ | not serious | very serious ^x^ | serious ^y^ | none | 110/6216 (1.8%) | 129/7161 (1.8%) | **RR 0.98** (-- to --) | **0 fewer per 1,000** (from -- to --) | ⨁◯◯◯ VERY LOW | CRITICAL |
| **Reduction in active TB incidence/prevalence in primary care - not measured** | | | | | | | | | | | | |
| - | - | - | - | - | - | - |  | | | | - |  |
| **Reduction in active TB incidence/prevalence in secondary/tertiary care ^ab^** | | | | | | | | | | | | |
| 2 ^7,8,t,u,v^ | observational studies | serious ^w^ | not serious | very serious ^x^ | serious ^y^ | none | 110/6216 (1.8%) | 129/7161 (1.8%) | **RR 0.98** (-- to --) | **0 fewer per 1,000** (from -- to --) | ⨁◯◯◯ VERY LOW | CRITICAL |

CI: Confidence interval; RR: Risk ratio

### Question 1: Explanations

a. PLEASE NOTE: The total number of studies measuring the effect of triage on the incidence of LTBI in all settings was 10. Four studies were excluded from the summary analysis (certainty estimates and crude summaries of findings [meta-analysis was NOT conducted]) because they did not report results in a format suitable for aggregation. These were (first author, year published): 1) Baussano, 2007; 2) Blumberg, 1998; 3) Louther, 1997; and 4) Yanai, 2003. Please see separate footnotes that summarise the results of these studies.

b. STUDY REPORTING OUTCOME BUT NOT INCLUDED IN SUMMARY ASSESSMENTS. Baussano, 2007: incidence rate of TST conversions of 106/4034 person-years before TBIC interventions were implemented, vs. 42 TST conversions per 4463 person-years after implementation (crude rate ratio 0.36 after vs. before).

c. STUDY REPORTING OUTCOME BUT NOT INCLUDED IN SUMMARY ASSESSMENTS. Blumberg, 1998 (some overlap with 1995 paper): TST conversion rate of 5.98/100 person-years in 1992 (pre-intervention) to 1.09/100 person-years from 1993–1997 (after the intervention was implemented; crude incidence rate ratio 0.18, after vs. before [derived from data presented]; authors report a p-value comparing the two time periods: <0.001).

d. STUDY REPORTING OUTCOME BUT NOT INCLUDED IN SUMMARY ASSESSMENTS. Yanai, 2003: TST conversions from 9.3 per 100 person-years (95% CI 3.3–15.3) before the implementation of TBIC measures (in 1995–1997) to 6.4 per 100 person-years (95% CI 1.5–11.4) and 2.2 per 100 person-years (95% CI 0–5.1), after implementation, in 1998 and 1999, respectively. Unadjusted rate ratio 0.9 (95% CI 0.4–2.2) for 1998 vs. 1995–1997 and 0.03 (95% CI 0.01–0.2) for 1999 vs. 1995–1997; adjusted rate ratio 0.4 (95% CI 0.1–1.6) and 0.01 (95% CI 0–0.04) for 1998 and 1999 vs. 1995–1997, respectively).

e. Definitions of triage varied widely between the six studies: Bangsberg - "all patients known HIV+, with HIV risk factors, or homelessness presenting with pneumonia/evidence of TB were isolated on presentation at the emergency room"; Blumberg 1995 - "expanded respiratory isolation policy"; Holzman - not defined; Roth - "rapid diagnosis and treatment"; Welbel - "revised policy (based on CDC guidelines) for isolation [CDC 1994: "in hospitals and other inpatient facilities, any patient suspected of having or known to have infectious TB should be placed in a TB isolation room"]; and Wenger - "higher index of suspicion for TB and stricter application of isolation criteria"

f. STUDY REPORTING OUTCOME BUT NOT INCLUDED IN SUMMARY ASSESSMENTS. Louther, 1997: 7.2 TST conversions per 100 person-years before the implementation of infection control measures, compared with 3.3 per 100 person-years after the implementation (crude rate ratio 0.46 [derived from data presented]; authors report p-value comparing the two groups: 0.001).

g. A mix of before/after, during/after, and prospective and retrospective cohort studies.

h. All studies are observational. Several studies have high risk of bias, with loss to follow-up, or incomplete ascertainment and/or reporting of outcomes of interest

i. Indirectness exists in the wide variation in types of triage and the descriptions of their implementation, as well as the implementation of a large number of infection control measures at one time. Please see assessment of directness for details.

j. Low number of events (<300) in almost all studies and two studies (Bangsberg and Wenger) have fewer than 20 events. The exception is the study by Roth et al., which has a total 2,878 events.

k. PLEASE NOTE: The total number of studies estimating the effect of triage on the incidence of LTBI in low TB burden settings was eight. Three studies were excluded from the summary analysis (certainty estimates and crude summaries of findings [meta-analysis was NOT conducted]) because they did not report results in a format suitable for aggregation. These were (first author, year published): 1) Baussano, 2007; 2) Blumberg, 1998; and 3) Louther, 1997. Please see separate footnotes that summarise the results of these studies.

l. Definitions of triage varied widely between the five studies: Bangsberg - "all patients known HIV+, with HIV risk factors, or homelessness presenting with pneumonia/evidence of TB were isolated on presentation at the emergency room"; Blumberg 1995 - "expanded respiratory isolation policy"; Holzman - not defined; Welbel - "revised policy (based on CDC guidelines) for isolation [CDC 1994: "in hospitals and other inpatient facilities, any patient suspected of having or known to have infectious TB should be placed in a TB isolation room"]; and Wenger - "higher index of suspicion for TB and stricter application of isolation criteria"

m. All studies have small numbers of events (<300; two had <20 events) and moderate overall sample sizes (except for Blumberg et al.)

n. PLEASE NOTE: The total number of studies estimating the effect of triage on the incidence of LTBI in high TB burden settings was two. One study was excluded from the summary analysis (certainty estimates and crude summaries of findings [meta-analysis was NOT conducted]) because it did not report results in a format suitable for aggregation. This was (first author, year published): 1) Yanai, 2003. Please see the separate footnote that summarises the results of this study.

o. High loss to follow-up.

p. Cannot comment on inconsistency as data from only one study included.

q. Very different definitions of triage used, population not well described, differences in background risk, and triage implemented along with other infection control measures. Please see assessment of directness for details.

r. PLEASE NOTE: The total number of studies measuring the effect of triage on the incidence of LTBI in secondary/tertiary care settings was 10. Four studies were excluded from the summary analysis (certainty estimates and crude summaries of findings [meta-analysis was NOT conducted]) because they did not report results in a format suitable for aggregation. These were (first author, year published): 1) Baussano, 2007; 2) Blumberg, 1998; 3) Louther, 1997; and 4) Yanai, 2003. Please see separate footnotes that summarise the results of these studies.

s. PLEASE NOTE: The total number of studies measuring the effect of triage on the incidence of TB disease in all settings was four. Two studies were excluded from the summary analysis (certainty estimates and crude summaries of findings [meta-analysis was NOT conducted]) because they did not report results in a format suitable for aggregation. These were (first author, year published): 1) Jacobson, 1957; and 2) O'Hara, 2017. Please see separate footnotes that summarise the results of these studies.

t. STUDY REPORTING OUTCOME BUT NOT INCLUDED IN SUMMARY ASSESSMENTS. Jacobson, 1957: incidence rate of 78 episodes of TB disease among healthcare workers in 38,331 person-years in the control group (1942–51, before the intervention was implemented) to 12 episodes in 18,229 person-years after the implementation of triage (1952–55; crude incidence rate ratio 0.32, after vs. before).

u. Definitions of triage differed between the two studies: Harries - "priority to patients with chronic cough; rapid collection of sputum specimens" and Yanai - "triage/isolation and expedited diagnosis training for health care workers"

v. STUDY REPORTING OUTCOME BUT NOT INCLUDED IN SUMMARY ASSESSMENTS. O'Hara, 2017: Unadjusted odds ratio (OR) for TB disease in HCWs at facilities with a higher administrative score was 0.94 (95% CI 0.87–1.02; p = 0.12). Adjusted OR (adjusted for environmental score, PPE score, miscellaneous score, and number of TB patients) 0.97 (95% CI 0.90–1.04; p = 0.36).

w. Under-ascertainment of outcomes in at least one study; poor reporting of loss to follow-up.

x. Very serious indirectness exists in terms of the population studied and the nature and implementation of the intervention. Please see assessment of directness for details.

y. Small numbers of events in both studies.

z. Small number of outcomes in before (n = 78) and after (n = 12) periods.

aa. PLEASE NOTE: The total number of studies measuring the effect of triage on the incidence of TB disease in high TB burden settings was three. One study was excluded from the summary analysis (certainty estimates and crude summaries of findings [meta-analysis was NOT conducted]) because it did not report results in a format suitable for aggregation. This was (first author, year published): 1) O'Hara, 2017. Please see the separate footnote that summarises the results of this study.

ab. PLEASE NOTE: The total number of studies measuring the effect of triage on the incidence of TB disease in secondary/tertiary care settings was four. Two studies were excluded from the summary analysis (certainty estimates and crude summaries of findings [meta-analysis was NOT conducted]) because they did not report results in a format suitable for aggregation. These were (first author, year published): 1) Jacobson, 1957; and 2) O'Hara, 2017. Please see separate footnotes that summarise the results of these studies.

### Question 1: References

1. Roth VR, Garrett DO, Laserson KF, Starling CE, Kritski AL, Medeiros EAS, Binkin N, Jarvis WR. A multicenter evaluation of tuberculin skin test positivity and conversion among health care workers in Brazilian hospitals. Int J Tuberc Lung Dis; 2005.

2. Wenger PN, Otten J, Breeden A, Orfas D, Beck-Sague CM, Jarvis WR. Control of nosocomial transmission of multidrug-resistant Mycobacterium tuberculosis among healthcare workers and HIV-infected patients. Lancet; 1995.

3. Welbel SF, French AL, Bush P, DeGuzman D,Weinstein RA. Protecting health care workers from tuberculosis: a 10-year experience. Am J Infect Control; 2009.

4. Blumberg HM, Watkins DL, Berschling JD, Antle A, Moore P, White N, Hunter M, Green B, Ray SM, McGowan Jr. J E. Preventing the nosocomial transmission of tuberculosis. Ann Intern Med; 1995.

5. Bangsberg DR, Crowley K, Moss A, Dobkin JF, McGregor C, Neu HC. Reduction in tuberculin skin-test conversions among medical house staff associated with improved tuberculosis infection control practices. Infect Control Hosp Epidemiol; 1997.

6. Holzman, RS. A comprehensive control program reduces transmission of tuberculosis to hospital staff. Clin Infect Dis; 1995.

7. Yanai H, Limpakarnjanarat K, Uthaivoravit W, Mastro TD, Mori T, Tappero JW. Risk of Mycobacterium tuberculosis infection and disease among health care workers, Chiang Rai, Thailand. Int J Tuberc Lung Dis; 2003.

8. Harries AD, Hargreaves NJ, Gausi F, Kwanjana JH, Salaniponi FM. Preventing tuberculosis among health workers in Malawi. Bull WHO; 2002.

9. Jacobson G, Hoyt DD, Bogen E. Tuberculosis in hospital employees as affected by an admission chest X-ray screening program. Dis Chest; 1957.

## Question 2: Can respiratory isolation (spatial separation) of presumed or demonstrated infectious TB cases reduce TB transmission to HCWs (including CHWs) when compared to transmission to HCWs (including CHWs) in settings with no intervention or different interventions?

Supplementary table 5. Question 2: Evidence summary

| **Certainty assessment** | | | | | | | **№ of patients** | | **Effect** | | **Certainty** | **Importance** |
| --- | --- | --- | --- | --- | --- | --- | --- | --- | --- | --- | --- | --- |
| **№ of studies** | **Study design** | **Risk of bias** | **Incon-sistency** | **Indirect-ness** | **Impre-cision** | **Other con-siderations** | **Respiratory isolation** | **No respiratory isolation** | **Relative (95% CI)** | **Absolute (95% CI)** |  |  |
| **Reduction in LTBI incidence/prevalence in all settings ^a^** | | | | | | | | | | | | |
| 12 ^1,2,3,4,5,6,7,8,9,10,11,12,b,c,d,e,f,g,h^ | observational studies | very serious ^i^ | not serious | very serious ^j^ | serious ^k^ | none | 2413/91397 (2.6%) | 1914/40097 (4.8%) | **RR 0.55** (-- to --) | **21 fewer per 1,000** (from -- to --) | ⨁◯◯◯ VERY LOW | CRITICAL |
| **Reduction in LTBI incidence/prevalence in low TB burden settings ^l^** | | | | | | | | | | | | |
| 11 ^1,2,4,5,6,7,8,9,10,11,12,b,c,d,f,h^ | observational studies | very serious ^m^ | not serious | very serious ^j^ | serious ^k^ | none | 653/88580 (0.7%) | 886/38495 (2.3%) | **RR 0.32** (-- to --) | **16 fewer per 1,000** (from -- to --) | ⨁◯◯◯ VERY LOW | CRITICAL |
| **Reduction in LTBI incidence/prevalence in high TB burden settings ^n^** | | | | | | | | | | | | |
| 1 ^3,e,g^ | observational studies | serious ^o^ | not serious ^p^ | serious ^j^ | not serious | none | 1760/2817 (62.5%) | 1028/1602 (64.2%) | **RR 0.97** (-- to --) | **19 fewer per 1,000** (from -- to --) | ⨁◯◯◯ VERY LOW | CRITICAL |
| **Reduction in LTBI incidence/prevalence in primary care - not measured** | | | | | | | | | | | | |
| - | - | - | - | - | - | - |  | | | | - |  |
| **Reduction in LTBI incidence/prevalence in secondary/tertiary care ^q^** | | | | | | | | | | | | |
| 12 ^1,2,3,4,5,6,7,8,9,10,11,12,b,c,d,e,f,g,h^ | observational studies | very serious ^i^ | not serious | very serious ^j^ | serious ^k^ | none | 2413/91397 (2.6%) | 1914/40097 (4.8%) | **RR 0.55** (-- to --) | **21 fewer per 1,000** (from -- to --) | ⨁◯◯◯ VERY LOW | CRITICAL |
| **Reduction in active TB incidence/prevalence in all settings ^r^** | | | | | | | | | | | | |
| 2 ^13,14,s,t^ | observational studies | serious ^u^ | not serious | very serious ^v^ | serious ^w^ | none | 110/6216 (1.8%) | 129/7161 (1.8%) | **RR 0.98** (-- to --) | **0 fewer per 1,000** (from -- to --) | ⨁◯◯◯ VERY LOW | CRITICAL |
| **Reductions in active TB incidence/prevalence in low TB burden settings - not measured** | | | | | | | | | | | | |
| - | - | - | - | - | - | - |  | | | | - |  |
| **Reductions in active TB incidence/prevalence in high TB burden settings ^x^** | | | | | | | | | | | | |
| 2 ^13,14,s,t^ | observational studies | serious ^u^ | not serious | very serious ^v^ | serious ^w^ | none | 110/6216 (1.8%) | 129/7161 (1.8%) | **RR 0.98** (-- to --) | **0 fewer per 1,000** (from -- to --) | ⨁◯◯◯ VERY LOW | CRITICAL |
| **Reductions in active TB incidence/prevalence in primary care** | | | | | | | | | | | | |
| 1 ^15,y^ | observational studies | very serious ^z^ | not serious ^p^ | very serious ^aa^ | serious ^ab^ | none |  |  | **OR 1.09** (0.99 to 1.19) | **1 fewer per 1,000** (from 1 fewer to 1 fewer) | ⨁◯◯◯ VERY LOW | CRITICAL |
| **Reductions in active TB incidence/prevalence in secondary/tertiary care ^ac^** | | | | | | | | | | | | |
| 2 ^13,14,t^ | observational studies | serious ^u^ | not serious | very serious ^v^ | serious ^w^ | none | 110/6216 (1.8%) | 129/7161 (1.8%) | **RR 0.98** (-- to --) | **0 fewer per 1,000** (from -- to --) | ⨁◯◯◯ VERY LOW | CRITICAL |

CI: Confidence interval; RR: Risk ratio; OR: Odds ratio

### Question 2: Explanations

a. PLEASE NOTE: The total number of studies measuring the effect of isolation on the incidence of LTBI in all settings was 19. Seven studies were excluded from the summary analysis (certainty estimates and crude summaries of findings [meta-analysis was NOT conducted]) because they did not report results in a format suitable for aggregation. These were (first author, year published): 1) Baussano, 2007; 2) Blumberg, 1998; 3) Bryan, 1983; 4) da Costa, 2009; 5) Louther, 1997; 6) Sinkowitz, 1996; and 7) Yanai, 2003. Please see separate footnotes that summarise the results of these studies.

b. STUDY REPORTING OUTCOME BUT NOT INCLUDED IN SUMMARY ASSESSMENTS. Baussano, 2007: incidence rate of TST conversions of 106/4034 person-years before TBIC interventions were implemented, vs. 42 TST conversions per 4463 person-years after implementation (crude rate ratio 0.36 after vs. before).

c. STUDY REPORTING OUTCOME BUT NOT INCLUDED IN SUMMARY ASSESSMENTS. Blumberg, 1998; some overlap with 1995 paper): TST conversion rate of 5.98/100 person-years in 1992 (pre-intervention) to 1.09/100 person-years from 1993-1997 (after the intervention was implemented; crude incidence rate ratio 0.18, after vs. before [derived from data presented]; authors report a p-value comparing the two time periods: <0.001).

d. STUDY REPORTING OUTCOME BUT NOT INCLUDED IN SUMMARY ASSESSMENTS. Bryan, 1983: TST conversion of 4.5% of HCWs in 1976, before the implementation of TBIC measures, vs. 5.1%, 1.5%, 0.85%, and 0.59% in the four years after implementation (crude risk ratio 1.13, 0.33, 0.19, and 0.13 for 1977–1981, respectively).

e. STUDY REPORTING OUTCOME BUT NOT INCLUDED IN SUMMARY ASSESSMENTS. da Costa, 2009: TST conversions incidence rate from 5.8 per 1,000 person-months (95% CI 4.9–6.7), to 3.7 per 1,000 person-months (95% CI 2.8–4.6); rate ratio 0.46 (95% CI 0.23–0.89) after vs. before, p = 0.006; adjusted rate ratio (adjusted for exposure and occupation) 0.24 (95% CI 0.10–0.54).

f. STUDY REPORTING OUTCOME BUT NOT INCLUDED IN SUMMARY ASSESSMENTS. Sinkowitz, 1996: TST conversion in 0%, 8.0%, and 5.1% of bronchoscopists in hospitals without IC measures and zero TB patients, 1–5 TB patients, and ≥6 TB patients, vs. 3.3%, 8.3%, and 5.7% in hospitals with the same numbers of TB patients but which had implemented four IC measures (crude risk ratio 1.04 and 1.12 [IC vs. no IC] for hospitals with 1–5 TB patients and ≥6 TB patients, respectively). In other HCWs, TST conversion in 0.49%, 0.64%, and 0.76% in hospitals without IC measures and zero TB patients, 1–5 TB patients, and 6 TB patients, vs. 0.53%, 0.69% and 0.90% in hospitals with the same numbers of TB patients but which had implemented four IC measures (crude risk ratio 1.08, 1.08, and 1.18 [IC vs. no IC] for hospitals with zero, 1–5 and ≥6 TB patients, respectively).

g. STUDY REPORTING OUTCOME BUT NOT INCLUDED IN SUMMARY ASSESSMENTS. Yanai, 2003: TST conversions from 9.3 per 100 person-years (95% CI 3.3–15.3) before the implementation of TBIC measures (in 1995–1997) to 6.4 per 100 person-years (95% CI 1.5–11.4) and 2.2 per 100 person-years (95% CI 0–5.1), after implementation, in 1998 and 1999, respectively. Unadjusted rate ratio 0.9 (95% CI 0.4–2.2) for 1998 vs. 1995–1997 and 0.03 (95% CI 0.01–0.2) for 1999 vs. 1995–1997; adjusted rate ratio 0.4 (95% CI 0.1–1.6) and 0.01 (95% CI 0–0.04) for 1998 and 1999 vs. 1995–1997, respectively).

h. STUDY REPORTING OUTCOME BUT NOT INCLUDED IN SUMMARY ASSESSMENTS. Louther, 1997: 7.2 TST conversions per 100 person-years before the implementation of infection control measures, compared with 3.3 per 100 person-years after the implementation (crude rate ratio 0.46 [derived from data presented]; authors report p-value comparing the two groups: 0.001).

i. Most studies included here have a high or unclear risk of bias. All are observational studies, some with high rates of loss to follow-up (e.g., Roth), low or unclear levels of participation, or incomplete reporting of outcomes (e.g., Blumberg). Two studies do not report results correctly or have missing results.

j. Indirectness was primarily through the implementation of multiple infection control measures together with isolation. Please see assessment of directness for details.

k. Imprecision exists: all except two studies (Fridkin and Roth) have fewer than 300 outcomes and three studies (Bangsberg, Behrman, and Wenger) have fewer than 20 outcomes.

l. PLEASE NOTE: The total number of studies measuring the effect of isolation on the incidence of LTBI in low TB burden settings was 16. Five studies were excluded from the summary analysis (certainty estimates and crude summaries of findings [meta-analysis was NOT conducted]) because they did not report results in a format suitable for aggregation. These were (first author, year published): 1) Baussano, 2007; 2) Blumberg, 1998; 3) Bryan, 1983; 4) Louther, 1997; and 5) Sinkowitz, 1996. Please see separate footnotes that summarise the results of these studies.

m. Most studies included here have a high or unclear risk of bias. All are observational studies, some have incomplete reporting of outcomes (e.g., Blumberg), and two studies do not report results correctly or have missing results.

n. PLEASE NOTE: The total number of studies measuring the effect of isolation on the incidence of LTBI in high TB burden settings was three. Two studies were excluded from the summary analysis (certainty estimates and crude summaries of findings [meta-analysis was NOT conducted]) because they did not report results in a format suitable for aggregation. These were (first author, year published): 1) da Costa, 2009 and 2) Yanai, 2003. Please see separate footnotes that summarise the results of these studies.

o. High proportions were lost to follow-up; those lost to follow-up may have been at higher risk of disease (more likely to be physicians).

p. Cannot comment on inconsistency as data from only one study are included.

q. PLEASE NOTE: The total number of studies measuring the effect of isolation on the incidence of LTBI in secondary/tertiary care settings was 19. Seven studies were excluded from the summary analysis (certainty estimates and crude summaries of findings [meta-analysis was NOT conducted]) because they did not report results in a format suitable for aggregation. These were (first author, year published): 1) Baussano, 2007; 2) Blumberg, 1998; 3) Bryan, 1983; 4) da Costa, 2009; 5) Louther, 1997; 6) Sinkowitz, 1996; and 7) Yanai, 2003. Please see separate footnotes that summarise the results of these studies.

r. PLEASE NOTE: The total number of studies measuring the effect of isolation on the incidence of active TB disease in all settings was four. Two studies were excluded from the summary analysis (certainty estimates and crude summaries of findings [meta-analysis was NOT conducted]) because they did not report results in a format suitable for aggregation. These were (first author, year published): 1) Claassens, 2013 and 2) O'Hara, 2017. Please see separate footnotes that summarise the results of these studies.

s. STUDY REPORTING OUTCOME BUT NOT INCLUDED IN SUMMARY ASSESSMENTS. Claassens, 2013: Unadjusted odds ratio for smear-positive TB among health care workers in facilities where administrative controls were implemented vs. facilities without (or with fewer) administrative controls 1.09 (95% CI 0.99–1.19), p = 0.07.

t. STUDY REPORTING OUTCOME BUT NOT INCLUDED IN SUMMARY ASSESSMENTS. O'Hara, 2017: Unadjusted odds ratio (OR) for TB disease in HCWs at facilities with a higher administrative score was 0.94 (95% CI 0.87–1.02; p = 0.12). Adjusted OR (adjusted for environmental score, PPE score, miscellaneous score, and number of TB patients) 0.97 (95% CI 0.90–1.04; p = 0.36).

u. Under-ascertainment of outcome in at least one study. All studies implemented isolation/spatial separation in addition to a number of other TBIC interventions; the effect of isolation/separation on the outcome of interest cannot be determined. Poor reporting of loss to follow-up.

v. Very serious indirectness exists, for populations studied and in the nature of and fidelity to the intervention. Please see assessment of directness for details.

w. Both studies had fewer than 200 events; one had fewer than 100 events.

x. PLEASE NOTE: The total number of studies measuring the effect of isolation on the incidence of active TB disease in high TB burden settings was four. Two studies were excluded from the summary analysis (certainty estimates and crude summaries of findings [meta-analysis was NOT conducted]) because they did not report results in a format suitable for aggregation. These were (first author, year published): 1) Claassens, 2013 and 2) O'Hara, 2017. Please see separate footnotes that summarise the results of these studies.

y. Please note that the odds ratio quoted for this study is for the development of smear-positive TB among healthcare workers at facilities classified by their implementation of infection control measures (i.e., the authors reported slightly increased odds of developing smear-positive TB in healthcare workers in facilities where administrative controls were implemented compared with facilities without or with fewer administrative controls).

z. High likelihood of under-ascertainment of outcome (smear-positive disease in HCWs), as only routine records used, without verification or any additional efforts to estimate numbers of cases. In addition, high variability in implementation intervention across different facilities, with isolation only implemented in ~50% of facilities. Most importantly, the study used the facilities as the base unit for assessing risk of TB disease (so reduced TB incidence to a binary of 'any' vs. 'no' HCWs developing TB at a particular facility) - individual HCW data not analysed.

aa. Indirectness is severe. Please see assessment of directness for details.

ab. Small effect seen, and in the opposite direction to expected. Confidence interval is narrow, but crosses 1.

ac. PLEASE NOTE: The total number of studies measuring the effect of isolation on the incidence of active TB disease in secondary/tertiary care settings was three. One study was excluded from the summary analysis (certainty estimates and crude summaries of findings [meta-analysis was NOT conducted]) because it did not report results in a format suitable for aggregation. This was (first author, year published): 1) O'Hara, 2017. Please see the separate footnote that summarises the results of this study.

### Question 2: References

1. Jones, SG. Evaluation of a human immunodeficiency virus rule out tuberculosis critical pathway as an intervention to decrease nosocomial transmission of tuberculosis in the inpatient setting. AIDS Patient Care Stds; 2002.

2. Jarvis, WR. Nosocomial transmission of multidrug-resistant Mycobacterium tuberculosis. Am J Infect Control; 1995.

3. Roth VR, Garrett DO, Laserson KF, Starling CE, Kritski AL, Medeiros EAS, Binkin N, Jarvis WR. A multicenter evaluation of tuberculin skin test positivity and conversion among health care workers in Brazilian hospitals.. Int J Tuberc Lung Dis; 2005.

4. Wenger PN, Otten J, Breeden A, Orfas D, Beck-Sague CM, Jarvis WR. Control of nosocomial transmission of multidrug-resistant Mycobacterium tuberculosis among healthcare workers and HIV-infected patients. Lancet; 1995.

5. Welbel SF, French AL, Bush P, DeGuzman D, Weinstein RA. Protecting health care workers from tuberculosis: a 10-year experience. Am J Infect Control; 2009.

6. Uyamadu N, Ahkee S, Carrico R, Tolentino A, Wojda B, Ramirez J. Reduction in tuberculin skin-test conversion rate after improved adherence to tuberculosis isolation. Infect Control Hosp Epidemiol; 1997.

7. Maloney SA, Pearson ML, Gordon MT, Del Castillo R, Boyle JF, Jarvis WR. Efficacy of control measures in preventing nosocomial transmission of multidrug-resistant tuberculosis to patients and health care workers. Ann Intern Med; 1995.

8. Fridkin SK, Manangan L, Bolyard E, Jarvis WR. SHEA-CDC TB survey, Part II: Efficacy of TB infection control programs at member hospitals, 1992. Society for Healthcare Epidemiology of America. Infect Control Hosp Epidemiol; 1995.

9. Blumberg HM, Watkins DL, Berschling JD, Antle A, Moore P, White N, Hunter M, Green B, Ray SM, McGowan Jr. J E. Preventing the nosocomial transmission of tuberculosis. Ann Intern Med; 1995.

10. Behrman AJ, Shofer FS. Tuberculosis exposure and control in an urban emergency department. Ann Emerg Med; 1998.

11. Bangsberg DR, Crowley K, Moss A, Dobkin JF, McGregor C, Neu HC. Reduction in tuberculin skin-test conversions among medical house staff associated with improved tuberculosis infection control practices. Infect Control Hosp Epidemiol; 1997.

12. Holzman, RS. A comprehensive control program reduces transmission of tuberculosis to hospital staff. Clin Infect Dis; 1995.

13. Yanai H, Limpakarnjanarat K, Uthaivoravit W, Mastro TD, Mori T, Tappero JW. Risk of Mycobacterium tuberculosis infection and disease among health care workers, Chiang Rai, Thailand. Int J Tuberc Lung Dis; 2003.

14. Harries AD, Hargreaves NJ, Gausi F, Kwanjana JH, Salaniponi FM. Preventing tuberculosis among health workers in Malawi. Bull WHO; 2002.

15. Claassens M, van Schalkwyk C, du Toit E, Roest E, Lombard CJ, Enarson DA, Beyers N, Borgdorff MW. Tuberculosis in Healthcare Workers and Infection Control Measures at Primary Healthcare Facilities in South Africa. PLoS One; 2013.

## Question 3: Can effective treatment of TB disease reduce TB transmission to HCWs (including CHWs) when compared to transmission to the same populations in settings where treatment is not yet administered?

Supplementary table 6. Question 3: Evidence summary

| **Certainty assessment** | | | | | | | **№ of patients** | | **Effect** | | **Certainty** | **Importance** |
| --- | --- | --- | --- | --- | --- | --- | --- | --- | --- | --- | --- | --- |
| **№ of studies** | **Study design** | **Risk of bias** | **Inconsistency** | **Indirectness** | **Imprecision** | **Other con-siderations** | **Effective treatment** | **No effective treatment** | **Relative (95% CI)** | **Absolute (95% CI)** |  |  |
| **Reduction in LTBI incidence/prevalence in all settings** | | | | | | | | | | | | |
| 4 ^1,2,3,4,a,b^ | observational studies | very serious ^c^ | serious ^d^ | very serious ^e^ | very serious ^f^ | none | 42/3081 (1.4%) | 155/3260 (4.8%) | **RR 0.29** (-- to --) | **34 fewer per 1,000** (from -- to --) | ⨁◯◯◯ VERY LOW | CRITICAL |
| **Reduction in LTBI incidence/prevalence in low TB burden settings** | | | | | | | | | | | | |
| 4 ^1,2,3,4,a,b^ | observational studies | very serious ^c^ | serious ^d^ | very serious ^e^ | very serious ^f^ | none | 42/3081 (1.4%) | 155/3260 (4.8%) | **RR 0.29** (-- to --) | **34 fewer per 1,000** (from -- to --) | ⨁◯◯◯ VERY LOW | CRITICAL |
| **Reduction in LTBI incidence/prevalence in high TB burden settings - not measured** | | | | | | | | | | | | |
| - | - | - | - | - | - | - |  | | | | - |  |
| **Reduction in LTBI incidence/prevalence in primary care - not measured** | | | | | | | | | | | | |
| - | - | - | - | - | - | - |  | | | | - |  |
| **Reduction in LTBI incidence/prevalence in secondary/tertiary care** | | | | | | | | | | | | |
| 4 ^1,2,3,4,a,b^ | observational studies | very serious ^c^ | serious ^d^ | very serious ^e^ | very serious ^f^ | none | 42/3081 (1.4%) | 155/3260 (4.8%) | **RR 0.29** (-- to --) | **34 fewer per 1,000** (from -- to --) | ⨁◯◯◯ VERY LOW | CRITICAL |
| **Reduction in active TB incidence/prevalence in all settings - not measured** | | | | | | | | | | | | |
| - | - | - | - | - | - | - |  | | | | - | CRITICAL |

CI: Confidence interval; RR: Risk ratio

### Question 3: Explanations

a. Please note that the study included by Welbel et al. does not describe, specifically, the implementation of treatment based on drug susceptibility, but only describes the introduction of drug susceptibility testing. We have assumed that the results of testing were then used to inform treatment.

b. Please note that meta-analysis was *not* conducted - pooled estimates and measures of effect are crude estimates.

c. There are design specific issues to these studies. Mainly, it is not possible to ascertain the effect of the intervention in question as the intervention is grouped with other interventions, which presents a serious risk of bias. There is also a serious design issue with the study by Wenger et al., as the intervention only differs slightly between before and after (3 agents vs. 4 agents). Though studies were not designed specifically to answer our question, the way they are designed does not give us confidence in the results of interest.

d. Some inconsistency exists. In the study by Jarvis, in particular, certain results are reported as unavailable, but the site of origin of these results is not specified, so this cannot be accounted for in analysis. In addition, in the study by Welbel et al., overall denominators for at-risk individuals are provided, but not the time period for which these individuals were at risk, reducing confidence in the estimates of risk.

e. Indirectness is severe and from many sources: population, intervention, and comparators (please see assessment of directness for details).

f. Serious imprecision exists. For a dichotomous outcome all studies have fewer than 110 cases (range 10–104). Samples sizes are also low in three studies (range 65–650; the exception is Welbel et al, with a sample size of 4,329).

### Question 3: References

1. Jarvis, WR. Nosocomial transmission of multidrug-resistant *Mycobacterium tuberculosis*. Am J Infect Control; 1995.

2. Wenger PN, Otten J, Breeden A, Orfas D, Beck-Sague CM, Jarvis WR. Control of nosocomial transmission of multidrug-resistant *Mycobacterium tuberculosis* among healthcare workers and HIV-infected patients. Lancet; 1995.

3. Welbel SF, French AL, Bush P, DeGuzman D, Weinstein RA. Protecting health care workers from tuberculosis: a 10-year experience. Am J Infect Control; 2009.

4. Maloney SA, Pearson ML, Gordon MT, Del Castillo R, Boyle JF, Jarvis WR. Efficacy of control measures in preventing nosocomial transmission of multidrug-resistant tuberculosis to patients and health care workers. Ann Intern Med; 1995.

## Question 4: Can triage of people with TB signs, symptoms reduce TB transmission to other persons attending healthcare settings when compared to transmission to the same populations in settings with no intervention or different interventions?

Supplementary table 7. Question 4: Evidence summary

| **Certainty assessment** | | | | | | | **№ of patients** | | **Effect** | | **Certainty** | **Importance** |
| --- | --- | --- | --- | --- | --- | --- | --- | --- | --- | --- | --- | --- |
| **№ of studies** | **Study design** | **Risk of bias** | **Inconsistency** | **Indirectness** | **Imprecision** | **Other consider-ations** | **Triage** | **No triage** | **Relative (95% CI)** | **Absolute (95% CI)** |  |  |
| **Reduction in LTBI incidence/prevalence in all settings - not measured** | | | | | | | | | | | | |
| - | - | - | - | - | - | - |  | | | | - |  |
| **Reduction in active TB incidence/prevalence in all settings** | | | | | | | | | | | | |
| 2 ^1,2,a^ | observational studies | serious ^b^ | not serious | very serious ^c^ | serious ^d^ | none | 5/237 (2.1%) | 45/306 (14.7%) | **RR 0.14** (-- to --) | **126 fewer per 1,000 (from -- to --)** | ⨁◯◯◯ VERY LOW | CRITICAL |
| **Reduction in active TB incidence/prevalence in low TB burden settings** | | | | | | | | | | | | |
| 2 ^1,2,a^ | observational studies | serious ^b^ | not serious | very serious ^c^ | serious ^d^ | none | 5/237 (2.1%) | 45/306 (14.7%) | **RR 0.14** (-- to --) | **126 fewer per 1,000 (from -- to --)** | ⨁◯◯◯ VERY LOW | CRITICAL |
| **Reduction in active TB incidence/prevalence in high TB burden settings - not measured** | | | | | | | | | | | | |
| - | - | - | - | - | - | - |  | | | | - |  |
| **Reduction in active TB incidence/prevalence in primary care - not measured** | | | | | | | | | | | | |
| - | - | - | - | - | - | - |  | | | | - |  |
| **Reduction in active TB incidence/prevalence in secondary/tertiary care** | | | | | | | | | | | | |
| 2 ^1,2,a^ | observational studies | serious ^b^ | not serious | very serious ^c^ | serious ^d^ | none | 5/237 (2.1%) | 45/306 (14.7%) | **RR 0.14** (-- to --) | **126 fewer per 1,000 (from -- to --)** | ⨁◯◯◯ VERY LOW | CRITICAL |
| **Reduction in active TB incidence/prevalence in HIV-negative individuals - not measured** | | | | | | | | | | | | |
| - | - | - | - | - | - | - |  | | | | - |  |
| **Reduction in active TB incidence/prevalence in HIV-positive individuals** | | | | | | | | | | | | |
| 2 ^1,2,a^ | observational studies | serious ^b^ | not serious | very serious ^c^ | serious ^d^ | none | 5/237 (2.1%) | 45/306 (14.7%) | **RR 0.14** (-- to --) | **126 fewer per 1,000 (from -- to --)** | ⨁◯◯◯ VERY LOW | CRITICAL |

CI: Confidence interval; RR: Risk ratio

### Question 4: Explanations

a. Please note that meta-analysis was *not* conducted - all summary estimates and measures of effect are crude estimates.

b. Serious risk of bias, probable to alter the results: exposure is different for each study between before and after groups; and not a clear differentiation of intervention vs. no intervention.

c. Multiple interventions were introduced at the same time. In addition, 'triage' was poorly defined in both studies, as targeting people with "respiratory disease and fever'" but with no mention of expedited diagnosis, or as an "increased index of suspicion for TB" without description of how this was implemented. Please see also assessment of directness.

d. Both studies had small sample sizes. The total at-risk population was 543; a total 50 events were included.

### Question 4: References

1. Stroud LA, Tokars JI, Grieco MH, Crawford JT, Culver DH, Edlin BR, Sordillo EM, Woodley CL, Gilligan ME, Schnieder N, Williams J, Jarvis WR. Evaluation of infection control measures in preventing the nosocomial transmission of multidrug-resistant *Mycobacterium tuberculosis* in a New York city hospital. Infect Control Hosp Epidemiol; 1995.

2. Moro ML, Errante I, Infuso A, Sodano L, Gori A, Orcese CA, Salamina G, D'Amico C, Besozii G, Caggese L. Effectiveness of infection control measures in controlling a nosocomial outbreak of multidrug-resistant tuberculosis among HIV patients in Italy. Int J Tuberc Lung Dis; 2000.

## Question 5: Can respiratory isolation (spatial separation) reduce TB transmission to other persons attending healthcare settings when compared to transmission to the same populations in settings with no intervention or different interventions?

Supplementary table 8. Question 5: Evidence summary

| **Certainty assessment** | | | | | | | **№ of patients** | | **Effect** | | **Certainty** | **Importance** |
| --- | --- | --- | --- | --- | --- | --- | --- | --- | --- | --- | --- | --- |
| **№ of studies** | **Study design** | **Risk of bias** | **Inconsist-ency** | **Indirect-ness** | **Impre-cision** | **Other consider-ations** | **Respiratory isolation** | **No respiratory isolation** | **Relative (95% CI)** | **Absolute (95% CI)** |  |  |
| **Reduction in LTBI incidence/prevalence in all settings - not measured** | | | | | | | | | | | | |
| - | - | - | - | - | - | - |  | | | | - |  |
| **Reduction in active TB incidence/prevalence in all settings** | | | | | | | | | | | | |
| 2 ^1,2,a^ | observational studies | serious ^b^ | not serious | very serious ^c^ | serious ^d^ | none | 5/237 (2.1%) | 45/306 (14.7%) | **RR 0.14** (-- to --) | **126 fewer per 1,000 (from -- to --)** | ⨁◯◯◯ VERY LOW | CRITICAL |
| **Reduction in active TB incidence/prevalence in low TB burden settings** | | | | | | | | | | | | |
| 2 ^1,2,a^ | observational studies | serious ^b^ | not serious | very serious ^c^ | serious ^d^ | none | 5/237 (2.1%) | 45/306 (14.7%) | **RR 0.14** (-- to --) | **126 fewer per 1,000 (from -- to --)** | ⨁◯◯◯ VERY LOW | CRITICAL |
| **Reduction in active TB incidence/prevalence in high TB burden settings - not measured** | | | | | | | | | | | | |
| - | - | - | - | - | - | - |  | | | | - |  |
| **Reduction in active TB incidence/prevalence in primary care - not measured** | | | | | | | | | | | | |
| - | - | - | - | - | - | - |  | | | | - |  |
| **Reduction in active TB incidence/prevalence in secondary/tertiary care** | | | | | | | | | | | | |
| 2 ^1,2,a^ | observational studies | serious ^b^ | not serious | very serious ^c^ | serious ^d^ | none | 5/237 (2.1%) | 45/306 (14.7%) | **RR 0.14** (-- to --) | **126 fewer per 1,000 (from -- to --)** | ⨁◯◯◯ VERY LOW | CRITICAL |
| **Reduction in active TB incidence/prevalence in HIV-negative individuals - not measured** | | | | | | | | | | | | |
| - | - | - | - | - | - | - |  | | | | - |  |
| **Reduction in active TB incidence/prevalence in HIV-positive individuals** | | | | | | | | | | | | |
| 2 ^1,2,a^ | observational studies | serious ^b^ | not serious | very serious ^c^ | serious ^d^ | none | 5/237 (2.1%) | 45/306 (14.7%) | **RR 0.14** (-- to --) | **126 fewer per 1,000 (from -- to --)** | ⨁◯◯◯ VERY LOW | CRITICAL |

CI: Confidence interval; RR: Risk ratio

### Question 5: Explanations

a. Please note that meta-analysis was *not* conducted - all summary estimates and measures of effect are crude estimates.

b. Serious risk of bias, probable to alter the results: exposure is different for each study between before and after groups; also isolation measures were in effect before and then more so after. Not a clear differentiation of intervention vs. no intervention.

c. Multiple interventions were introduced at the same time.

d. Both studies had small sample sizes. The total at-risk population was 543; a total 50 events were included.

### Question 5: References

1. Moro ML, Errante I, Infuso A, Sodano L, Gori A, Orcese CA, Salamina G, D'Amico C, Besozii G, Caggese L. Effectiveness of infection control measures in controlling a nosocomial outbreak of multidrug-resistant tuberculosis among HIV patients in Italy. Int J Tuberc Lung Dis; 2000.

2. Stroud LA, Tokars JI, Grieco MH, Crawford JT, Culver DH, Edlin BR, Sordillo EM, Woodley CL, Gilligan ME, Schnieder N, Williams J, Jarvis WR. Evaluation of infection control measures in preventing the nosocomial transmission of multidrug-resistant *Mycobacterium tuberculosis* in a New York city hospital. Infect Control Hosp Epidemiol; 1995.

## Question 6: Can effective treatment of TB disease reduce TB transmission to other persons attending healthcare settings when compared to transmission to the same populations in settings where treatment administration is delayed?

Supplementary table 9. Question 6: Evidence summary

| **Certainty assessment** | | | | | | | **№ of patients** | | **Effect** | | **Certainty** | **Importance** |
| --- | --- | --- | --- | --- | --- | --- | --- | --- | --- | --- | --- | --- |
| **№ of studies** | **Study design** | **Risk of bias** | **Inconsistency** | **Indirectness** | **Imprecision** | **Other consider-ations** | **Effective treatment** | **No effective treatment** | **Relative (95% CI)** | **Absolute (95% CI)** |  |  |
| **Reduction in LTBI incidence/prevalence in all settings - not measured** | | | | | | | | | | | | |
| - | - | - | - | - | - | - |  | | | | - |  |
| **Reduction in active TB incidence/prevalence in all settings** | | | | | | | | | | | | |
| 1 ^1,a^ | observational studies | serious ^b^ | not serious ^c^ | very serious ^d^ | serious ^e^ | none | 5/193 (2.6%) | 19/216 (8.8%) | **RR 0.29** (-- to --) | **62 fewer per 1,000 (from -- to --)** | ⨁◯◯◯ VERY LOW | CRITICAL |
| **Reduction in active TB incidence/prevalence in low TB burden settings** | | | | | | | | | | | | |
| 1 ^1,a^ | observational studies | serious ^b^ | not serious ^c^ | very serious ^d^ | serious ^e^ | none | 5/193 (2.6%) | 19/216 (8.8%) | **RR 0.29** (-- to --) | **62 fewer per 1,000 (from -- to --)** | ⨁◯◯◯ VERY LOW | CRITICAL |
| **Reduction in active TB incidence/prevalence in high TB burden settings - not measured** | | | | | | | | | | | | |
| - | - | - | - | - | - | - |  | | | | - |  |
| **Reduction in active TB incidence/prevalence in primary care - not measured** | | | | | | | | | | | | |
| - | - | - | - | - | - | - |  | | | | - |  |
| **Reduction in active TB incidence/prevalence in secondary/tertiary care** | | | | | | | | | | | | |
| 1 ^1,a^ | observational studies | serious ^b^ | not serious ^c^ | very serious ^d^ | serious ^e^ | none | 5/193 (2.6%) | 19/216 (8.8%) | **RR 0.29** (-- to --) | **62 fewer per 1,000 (from -- to --)** | ⨁◯◯◯ VERY LOW | CRITICAL |
| **Reduction in active TB incidence/prevalence in HIV-negative individuals - not measured** | | | | | | | | | | | | |
| - | - | - | - | - | - | - |  | | | | - |  |
| **Reduction in active TB incidence/prevalence in HIV-positive individuals** | | | | | | | | | | | | |
| 1 ^1,a^ | observational studies | serious ^b^ | not serious ^c^ | very serious ^d^ | serious ^e^ | none | 5/193 (2.6%) | 19/216 (8.8%) | **RR 0.29** (-- to --) | **62 fewer per 1,000 (from -- to --)** | ⨁◯◯◯ VERY LOW | CRITICAL |

CI: Confidence interval; RR: Risk ratio

### Question 6: Explanations

a. Please note that meta-analysis was *not* conducted - all summary estimates and measures of effect are crude estimates.

b. No significant difference in the treatment in the before and after groups (1.5 vs. 2.0 drugs given before vs. after; range 0-4 in both periods; p = 0.2). Exposure is also different for between before and after groups.

c. As there is only one study included we cannot comment on heterogeneity of results between studies.

d. Authors describe "expanded use of antituberculous drugs" in 'after' period, but no description of time to treatment; therefore unable to assess for difference compared with delayed treatment administration.

e. Small numbers of cases in both arms. Overall number of exposed individuals = 409 (n = 216 before; n = 193 after)

### Question 6: References

1. Stroud LA, Tokars JI, Grieco MH, Crawford JT, Culver DH, Edlin BR, Sordillo EM, Woodley CL, Gilligan ME, Schnieder N, Williams J, Jarvis WR. Evaluation of infection control measures in preventing the nosocomial transmission of multidrug-resistant *Mycobacterium tuberculosis* in a New York city hospital. Infect Control Hosp Epidemiol; 1995.

# Appendix 4: PRISMA* checklist

| **Section/topic** | **#** | **Checklist item** | **Reported on** |
| --- | --- | --- | --- |
| **TITLE** | | |  |
| **Title** | **1** | Identify the report as a systematic review, meta-analysis, or both. | **Page 1** |
| **ABSTRACT** | | |  |
| **Structured summary** | **2** | Provide a structured summary including, as applicable: background; objectives; data sources; study eligibility criteria, participants, and interventions; study appraisal and synthesis methods; results; limitations; conclusions and implications of key findings; systematic review registration number. | **Pages 2 and 3** |
| **INTRODUCTION** | | |  |
| **Rationale** | **3** | Describe the rationale for the review in the context of what is already known. | **Page 4** |
| **Objectives** | **4** | Provide an explicit statement of questions being addressed with reference to participants, interventions, comparisons, outcomes, and study design (PICOS). | **Page 4** |
| **METHODS** | | |  |
| **Protocol and registration** | **5** | Indicate if a review protocol exists, if and where it can be accessed (e.g., Web address), and, if available, provide registration information including registration number. | **Page 4** |
| **Eligibility criteria** | **6** | Specify study characteristics (e.g., PICOS, length of follow-up) and report characteristics (e.g., years considered, language, publication status) used as criteria for eligibility, giving rationale. | **Page 5, Table 1, and Appendix 1** |
| **Information sources** | **7** | Describe all information sources (e.g., databases with dates of coverage, contact with study authors to identify additional studies) in the search and date last searched. | **Page 5 and Appendix 1 (Supplementary table 2)** |
| **Search** | **8** | Present full electronic search strategy for at least one database, including any limits used, such that it could be repeated. | **Supplementary table 1** |
| **Study selection** | **9** | State the process for selecting studies (i.e., screening, eligibility, included in systematic review, and, if applicable, included in the meta-analysis). | **Page 5, Table 1, and Appendix 1** |
| **Data collection process** | **10** | Describe method of data extraction from reports (e.g., piloted forms, independently, in duplicate) and any processes for obtaining and confirming data from investigators. | **Page 5 and Appendix 1** |
| **Data items** | **11** | List and define all variables for which data were sought (e.g., PICOS, funding sources) and any assumptions and simplifications made. | **Appendix 1** |
| **Risk of bias in individual studies** | **12** | Describe methods used for assessing risk of bias of individual studies (including specification of whether this was done at the study or outcome level), and how this information is to be used in any data synthesis. | **Page 6** |
| **Summary measures** | **13** | State the principal summary measures (e.g., risk ratio, difference in means). | **N/A** – findings were synthesised in a narrative; meta-analysis was not feasible due to heterogeneity in the way data were reported. |
| **Synthesis of results** | **14** | Describe the methods of handling data and combining results of studies, if done, including measures of consistency (e.g., I^2^) for each meta-analysis. | **N/A** – findings were synthesised in a narrative; meta-analysis was not feasible due to heterogeneity in the way data were reported. |
| **Risk of bias across studies** | **15** | Specify any assessment of risk of bias that may affect the cumulative evidence (e.g., publication bias, selective reporting within studies). | **Page 7** |
| **Additional analyses** | **16** | Describe methods of additional analyses (e.g., sensitivity or subgroup analyses, meta-regression), if done, indicating which were pre-specified. | **N/A** |
| **RESULTS** | | |  |
| **Study selection** | **17** | Give numbers of studies screened, assessed for eligibility, and included in the review, with reasons for exclusions at each stage, ideally with a flow diagram. | **Page 7** for text; **Figure 1** (page 8) for flow diagram; **Table 2** (page 9) for summary characteristics of studies included; **Supporting table 3** for list of included systematic reviews and guidelines |
| **Study characteristics** | **18** | For each study, present characteristics for which data were extracted (e.g., study size, PICOS, follow-up period) and provide the citations. | **Table 2** (page 9) of summary characteristics of studies included; **Table 3** (pages 11–18) for setting, design, population(s) studied, intervention(s) implemented, and outcome(s) measured in the studies included; **Table 4** (pages 20–27) for main findings of studies included |
| **Risk of bias within studies** | **19** | Present data on risk of bias of each study and, if available, any outcome level assessment (see item 12). | **Page 29** for text; **Table 5** (page 29) for summary of quality assessments for retrospective studies; **Table 6** (page 30) for summary of quality assessment for prospective studies |
| **Results of individual studies** | **20** | For all outcomes considered (benefits or harms), present, for each study: (a) simple summary data for each intervention group (b) effect estimates and confidence intervals, ideally with a forest plot. | **N/A** – findings were synthesised in a narrative; meta-analysis was not feasible due to heterogeneity in the way data were reported. |
| **Synthesis of results** | **21** | Present results of each meta-analysis done, including confidence intervals and measures of consistency. | **N/A** – findings were synthesised in a narrative; meta-analysis was not feasible due to heterogeneity in the way data were reported. |
| **Risk of bias across studies** | **22** | Present results of any assessment of risk of bias across studies (see Item 15). | **Supplementary tables 4–9** (GRADE assessment) |
| **Additional analysis** | **23** | Give results of additional analyses, if done (e.g., sensitivity or subgroup analyses, meta-regression [see Item 16]). | **N/A** |
| **DISCUSSION** | | |  |
| **Summary of evidence** | **24** | Summarize the main findings including the strength of evidence for each main outcome; consider their relevance to key groups (e.g., healthcare providers, users, and policy makers). | **Pages 30–32** |
| **Limitations** | **25** | Discuss limitations at study and outcome level (e.g., risk of bias), and at review-level (e.g., incomplete retrieval of identified research, reporting bias). | **Page 34** |
| **Conclusions** | **26** | Provide a general interpretation of the results in the context of other evidence, and implications for future research. | **Page 34** |
| **FUNDING** | | |  |
| **Funding** | **27** | Describe sources of funding for the systematic review and other support (e.g., supply of data); role of funders for the systematic review. | **Page 35** |

*From: Moher D, Liberati A, Tetzlaff J, Altman DG, The PRISMA Group (2009). Preferred Reporting Items for Systematic Reviews and Meta-Analyses: The PRISMA Statement. PLoS Med 6(6): e1000097. doi:10.1371/journal.pmed1000097

# Appendix 5: Additional references

1 Falconer J. Search strategies for “Evidence for the use of administrative controls to reduce the transmission of Mycobacterium tuberculosis in healthcare settings: a systematic review.” LSHTM Data Compass. London, United Kingdom: London School of Hygiene & Tropical Medicine. 2017. https://datacompass.lshtm.ac.uk/1020/ (accessed 2019 Mar 18)

2 World Health Organization. Use of high burden country lists for TB by WHO in the post-2015 era. 2016. http://www.who.int/tb/publications/global_report/high_tb_burdencountrylists2016-2020.pdf?ua=1 (accessed 2018 Mar 2)

3 Schmidt B-M, Engel ME, Abdullahi L, Ehrlich R. Effectiveness of control measures to prevent occupational tuberculosis infection in health care workers: a systematic review. *BMC Public Health*. 2018;**18**(1):661.

4 Nathavitharana RR, Bond P, Dramowski A, et al. Agents of change: The role of healthcare workers in the prevention of nosocomial and occupational tuberculosis. *Press Medicale*. 2017;**46**(2 Pt 2):e53–62.

5 Nasreen S, Shokoohi M, Malvankar-Mehta MS. Prevalence of Latent Tuberculosis among Health Care Workers in High Burden Countries: A Systematic Review and Meta-Analysis. *PLoS One*. 2016;**11**(10):e0164034.

6 Punjabi CD, Perloff SR, Zuckerman JM. Preventing Transmission of Mycobacterium tuberculosis in Health Care Settings. *Infect Dis Clin North Am*. 2016;**30**(4):1013–22.

7 Van Cutsem G, Isaakidis P, Farley J, Nardell E, Volchenkov G, Cox H. Infection Control for Drug-Resistant Tuberculosis: Early Diagnosis and Treatment Is the Key. *Clin Infect Dis*. 2016;**62**:S238–43.

8 Verkuijl S, Middelkoop K. Protecting Our Front-liners: Occupational Tuberculosis Prevention Through Infection Control Strategies. *Clin Infect Dis*. 2016;**62**:S231–7.

9 Zuckerman JM. Prevention of health care-acquired pneumonia and transmission of Mycobacterium tuberculosis in health care settings. *Infect Dis Clin North Am*. 2011;**25**(1):117–33.

10 Trajman A, Menzies D. Occupational respiratory infections. *Curr Opin Pulm Med*. 2010;**16**(3):226–34.

11 Conde MB, Melo FAF de, Marques AMC, et al. III Brazilian Thoracic Association Guidelines on tuberculosis. *J Bras Pneumol*. 2009;**35**(10):1018–48.

12 World Health Organization. WHO Policy on TB Infection Control in Health-Care Facilities, Congregate Settings and Households. 2009. http://www.who.int/tb/publications/2009/infection_control/en/ (accessed 2018 Mar 22)

13 Humphreys H. Control and prevention of healthcare-associated tuberculosis: the role of respiratory isolation and personal respiratory protection. *J Hosp Infect*. 2007;**66**(1):1–5.

14 Menzies D, Joshi R, Pai M. Risk of tuberculosis infection and disease associated with work in health care settings. *Int J Tuberc Lung Dis*. 2007;**11**(6):593–605.

15 Joshi R, Reingold AL, Menzies D, Pai M. Tuberculosis among health-care workers in low- and middle-income countries: a systematic review. *PLoS Med / Public Libr Sci*. 2006;**3**(12):e494–e494.

16 Tam CM, Leung CC. Occupational tuberculosis: a review of the literature and the local situation. *Hong Kong Med J*. 2006;**12**(6):448–55.

17 Jensen PA, Lambert LA, Iademarco MF, Ridzon R, CDC. Guidelines for preventing the transmission of Mycobacterium tuberculosis in health-care settings, 2005. *MMWR Recomm reports Morb Mortal Wkly report Recomm reports*. 2005;**54**(RR-17):1–141.

18 Sepkowitz KA. Tuberculosis control in the 21st century. *Emerg Infect Dis*. 2001;**7**(2):259–62.

19 Raymond L. ACOEM guidelines for protecting health care workers against tuberculosis. American College of Occupational and Environmental Medicine. *J Occup Environ Med*. 1998;**40**(9):765–7.

20 Cookson ST, Jarvis WR. Prevention of nosocomial transmission of Mycobacterium tuberculosis. *Infect Dis Clin North Am*. 1997;**11**(2):385–409.

21 Davis YM, McCray E, Simone PM. Hospital infection control practices for tuberculosis. *Clin Chest Med*. 1997;**18**(1):19–33.

22 Harries AD, Maher D, Nunn P. Practical and affordable measures for the protection of health care workers from tuberculosis in low-income countries. *Bull World Health Org*. 1997;**75**(5):477–89.

23 Members of the Ad Hoc Committee for the Guidelines for Preventing the Transmission fo Tuberculosis in Canadian Health Care F, Other Institutional S. Guidelines for preventing the transmission of tuberculosis in Canadian Health Care Facilities and other institutional settings. *Canada Commun Dis Rep*. 1996;**22 Suppl 1**:i–iv, 1-50, i–iv, 1-55.

24 L’Ecuyer PB, Fraser VJ. Further progress in the portection of healthcare workers. *Curr Opin Infect Dis*. 1996;**9**:280–5.

25 McGowan JEJ. Nosocomial tuberculosis: new progress in control and prevention. *Clin Infect Dis*. 1995;**21**(3):489–505.

26 Menzies D, Fanning A, Yuan L, Fitzgerald M. Tuberculosis among health care workers. *N Engl J Med*. 1995;**332**(2):92–8.

27 Seyferth PD. Guidelines for preventing occupational exposure to TB. *Missouri Dent J*. 1994;**74**(4):10–1.
